# The quest for mammalian Polycomb response elements: are we there yet?

**DOI:** 10.1007/s00412-015-0539-4

**Published:** 2015-10-09

**Authors:** Moritz Bauer, Johanna Trupke, Leonie Ringrose

**Affiliations:** Institute of Molecular Biotechnology (IMBA), Dr. Bohr-Gasse 3, 1030 Vienna, Austria; IRI Life Sciences, Humboldt Universität zu Berlin, Philippstr. 13, Haus 18, 10115 Berlin, Germany

**Keywords:** Polycomb, Trithorax, Polycomb response element, Mammal, Drosophila, Epigenetics

## Abstract

A long-standing mystery in the field of Polycomb and Trithorax regulation is how these proteins, which are highly conserved between flies and mammals, can regulate several hundred equally highly conserved target genes, but recognise these targets via *cis*-regulatory elements that appear to show no conservation in their DNA sequence. These elements, termed Polycomb/Trithorax response elements (PRE/TREs or PREs), are relatively well characterised in flies, but their mammalian counterparts have proved to be extremely difficult to identify. Recent progress in this endeavour has generated a wealth of data and raised several intriguing questions. Here, we ask why and to what extent mammalian PREs are so different to those of the fly. We review recent advances, evaluate current models and identify open questions in the quest for mammalian PREs.

**Electronic supplementary material:**

The online version of this article (doi:10.1007/s00412-015-0539-4) contains supplementary material, which is available to authorized users.

## Introduction: the central mystery of fly and vertebrate PREs

The highly conserved Polycomb (PcG) and Trithorax (TrxG) group proteins work antagonistically on several hundred developmentally important target genes, to maintain repressed (PcG) or active (TrxG) transcription states (Simon and Kingston 2013; Steffen and Ringrose 2014). The repertoire of target genes that are regulated by the PcG and TrxG is remarkably similar in flies and vertebrates, including the *Hox* genes, many master transcriptional regulators, and genes involved in signalling and proliferation (Ringrose 2007). Given the identity of these targets, it is not surprising that aberrant expression of the PcG and TrxG proteins can lead to developmental defects and cancer (Kennison 2004; Richly et al. 2011). However, given the similarity of the target genes, together with the high conservation of the PcG and TrxG proteins themselves, it is extremely intriguing that the DNA sequences to which they bind show no apparent similarity between flies and vertebrates (Kassis and Brown 2013; Ringrose and Paro 2007).

In flies, the PcG and TrxG proteins act through Polycomb/Trithorax response elements (PREs). Whilst the properties of PREs and the DNA sequences that define them are reasonably well characterised in flies, the analogous elements in mammals have proved highly elusive (Kassis and Brown 2013; Steffen and Ringrose 2014). The effort to identify and understand mammalian PREs is currently one of the most active and controversial areas in the PcG/TrxG field.

Understanding the design principles and functions of mammalian PREs will be crucial for understanding genome-wide mammalian PcG/TrxG function in health and disease. Why are PREs not conserved? Do mammalian PREs use different sequences but perform essentially the same function as fly PREs? Or does mammalian PcG/TrxG regulation play by fundamentally different rules to those in the fly? In this review, we address these questions and evaluate recent progress in the quest for mammalian PREs.

## What makes a fly PRE? Different properties depend on context

PREs are best characterised in flies. These fascinating *cis*-regulatory elements work in concert with enhancers to ensure genome-wide transcriptional fidelity; however, PREs are distinct from enhancers in two key aspects. First, whereas enhancers respond to the cellular concentrations of transcription factors with exquisite precision in different cell types, PREs do not depend on the cellular concentrations of the PcG and TrxG proteins, which are ubiquitously expressed. Instead, PREs can adopt an active or silent state by responding to the status of their associated enhancers and promoters (Maeda and Karch 2006). Second, whereas enhancers can determine patterns of gene transcription, PREs alone cannot do so. However, PREs can maintain the transcriptional status that has initially been determined by transcription factors acting at enhancers. This maintenance can persist over many cell generations, even in the absence of the initial determining transcription factors (Chan et al. 1994). Thus, PREs can give stable epigenetic memory of both silenced and active transcriptional states (reviewed in Steffen and Ringrose 2014). However, despite this stability, PREs also have a built-in flexibility, allowing switching or modulation of their output in response to developmental, environmental or metabolic cues (Cavalli and Paro 1998; Herzog et al. 2014; Ost et al. 2014).

In summary, PREs as we know them from the fly can fulfil four tasks: first, they recruit PcG and TrxG proteins, and second, establish an active or silent state depending on inputs from their associated promoter and enhancer. Third, the PRE may maintain a memory of this state, and fourth, it may switch states upon new incoming signals. There is accumulating evidence that each of these four properties can be quantitatively different in different developmental contexts. For example, one PRE can be switched early in development but not later (Cavalli and Paro 1998), and another is biased towards activation in early development but prefers silencing at later stages (Herzog et al. 2014). Furthermore, it is becoming clear that different fly PREs have different inherent “personalities”. Just as some people have an excellent memory and others continuously forget things, different PREs have different abilities in each of the four tasks outlined above (Beuchle et al. 2001; Okulski et al. 2011). These differences are likely to be fundamentally important for the regulation of their target genes. Fly PREs are composed of multiple short DNA motifs, whose number and order is highly variable from one PRE to another (Ringrose and Paro 2007; Kassis and Brown 2013), and also varies for the same PRE across different fly species (Hauenschild et al. 2008). To fully understand the “PRE code”, it will be essential to understand how DNA sequence modulates PRE output via interaction of the PRE with PcG and TrxG proteins, the surrounding genomic landscape and incoming signals, to understand why different elements have different quantitative responses to specific inputs.

## What makes a mammalian PRE? Do they have analogous functions to fly PREs?

Given this situation in the fly, where do we stand with mammalian PREs? The first fly PREs were discovered over 20 years ago (Chan et al. 1994; Kassis 1994; Simon et al. 1993), whereas the first mammalian PREs were described only 5 years ago (Sing et al. 2009; Woo et al. 2010). Since then, the vast majority of work in the mammalian field has focused on defining DNA elements that can fulfil the first task of PREs, namely to recruit PcG proteins (little attention has focused on TrxG recruitment). What is the evidence that mammalian PREs can or need to perform the other functions shown by fly PREs? To answer this question, we review similarities and differences between flies and vertebrates in the different components of this regulatory system, namely the PcG and TrxG proteins, their target genes, and the mammalian PREs defined so far. For each component, we ask whether these similarities and differences throw light on what makes a mammalian PRE.

We will not cover the recent large body of work on the involvement of 3D genome architecture in PcG and TrxG function, since large-scale spatial events occur downstream of the initial targeting of PcG and TrxG proteins to PREs. In addition, there is emerging evidence that these long-range interactions may be mediated by insulator elements rather than PREs themselves. These topics have been covered in detail in several recent reviews (Pirrotta and Li 2012; Noordermeer and Duboule 2013; Smigova et al. 2014; Cheutin and Cavalli 2014).

## Fly and vertebrate PcG and TrxG proteins

### PcG complexes: conserved at the core, with higher mammalian diversity

**Fig. 1:**
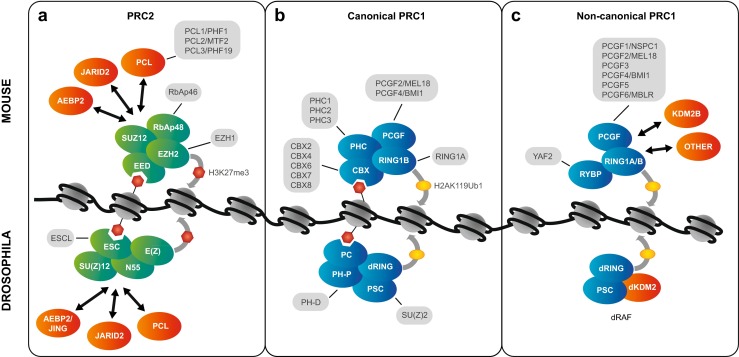
Composition of PcG Complexes in flies and vertebrates. The Polycomb repressive complex 2 (PRC2) and Polycomb repressive complex 1 (PRC1) family of complexes are shown. Core subunits are shown in *green* for PRC2 and *blue* for PRC1. Alternate subunits, derived from multiple genes and if more than two, are shown in *grey*. Accessory proteins are shown in *orange. Top*: mouse complexes; *bottom*: *Drosophila* complexes. Selected histone modifications are shown: *Red hexagons*: histone H3 tail trimethylated at lysine 27 (H3K27me3); *yellow ovals*: histone H2A monoubiquitinated at lysine 119 (vertebrates) or 118 (fly). (H2AK119/118 Ub). **a** PRC2 consists of four core subunits, EZH1/2 (fly E(Z)), EED (fly ESC), SUZ12 (fly SU(Z)12), and RbAp46/48 (or RBBP7/4; fly NURF55) (Cao et al. 2002; Czermin et al. 2002; Kuzmichev et al. 2002; Muller et al. 2002), and three accessory proteins, PCL (Walker et al. 2010), JARID2 (Herz et al. 2012; Kalb et al. 2014; Landeira et al. 2010; Li et al. 2010; Pasini et al. 2010a; Peng et al. 2009; Shen et al. 2009), and AEBP2 (Cao and Zhang 2004; Kalb et al. 2014). Alternate translation start site usage results in four different EED isoforms (not shown in the figure), which have different preferred histone substrates (Kuzmichev et al. 2004). PRC2 dimethylates and trimethylates histone H3 at Lys27 (H3K27me3) through the SET domain of EZH1/2 (fly E(Z)) (Cao et al. 2002; Czermin et al. 2002; Kuzmichev et al. 2002; Muller et al. 2002). In addition, PRC2 can bind H3K27me3 via EED (Hansen et al. 2008; Margueron et al. 2009). **b** Canonical PRC1 consists of four core subunits, RING1A/B (fly dRING), CBX (fly PC), PCGF (fly PSC or SU(Z)2), and PHC (fly PH) (Gil and O’Loghlen 2014; Simon and Kingston 2009). PRC1 catalyses H2AK119Ub1 (in flies H2AK118Ub1) through its RING1A/B (fly dRING) subunit (Cao et al. 2005; de Napoles et al. 2004; Scheuermann et al. 2012; Wang et al. 2004a). Canonical PRC1 can bind H3K27me3 via the chromodomain of CBX2 or 7 (fly PC) (Bernstein et al. 2006b; Fischle et al. 2003; Min et al. 2003); however, different CBX proteins have different preferences for modified histone tails (Bernstein et al. 2006b), see main text and Fig. 2 for details. **c** *Top*: one class of vertebrate non-canonical PRC1s consists of three core subunits, RING1A/B, PCGF, and RYBP or YAF2 and various accessory proteins. The complexes are distinguished by different PCGF subunits. The complex containing PCGF1 (PRC1.1) also contains the histone H3K36 demethylase KDM2B. Other PCGF subunits copurify with other accessory proteins (*orange*) (Gao et al. 2012). *Bottom*: *Drosophila* dRAF is the most similar to vertebrate PRC1.1 and consists of dRING, PSC, and the histone H3K36 demethylase dKDM2 (Lagarou et al. 2008). Further non-canonical PRC1s exist and are reviewed in Gil and O’Loghlen (2014) and Simon and Kingston (2013). See main text and Table 1 for detail on molecular properties

The core components of PcG complexes are remarkably conserved between flies and vertebrates (Fig. 1). However, the most striking difference is that vertebrate complexes make use of multiple alternative versions of some subunits, which can be deployed at different developmental stages, at different genomic locations, and can confer different properties on the complex (Gil and O’Loghlen 2014; Margueron and Reinberg 2011; Simon and Kingston 2013). This in turn may place different requirements on the PREs that recruit them.

Two key PcG complexes are Polycomb repressive complex 2 (PRC2, Fig. 1a) and Polycomb repressive complex 1 (PRC1, Fig. 1b). Both at the amino acid sequence level and at the level of subunit diversity, PRC2 is more evolutionarily conserved than PRC1 (reviewed in Margueron and Reinberg 2011; Ringrose and Paro 2004; Schuettengruber et al. 2007). In fly PRC2, a single subunit, enhancer of zeste (E(Z)), is used throughout development, mediating dimethylation and trimethylation of histone H3 on lysine 27 (H3K27me2/3) (Czermin et al. 2002; Muller et al. 2002). In contrast, in mammalian PRC2, this role is taken by the EZH2 or EZH1 subunit. These two closely related proteins have markedly different activities and expression patterns. EZH2 has similar catalytic activity to fly E(Z) (Cao et al. 2002; Kuzmichev et al. 2002) and is predominantly found in embryonic stem cells (ESCs) and proliferating cells, whereas EZH1 replaces EZH2 in specific differentiating and non-dividing cell types (Margueron et al. 2008; Shen et al. 2008; Stojic et al. 2011). The enzymatic activity of EZH1 appears to be context dependent: it has been reported to show similar activity to EZH2 in vitro (Shen et al. 2008), to have reduced activity in vivo (Margueron et al. 2008), and in some cases to promote transcriptional activation (Mousavi et al. 2012; Xu et al. 2015). Whether the enzymatic activity of fly E(Z) is modulated in specific cell lineages or at specific target genes to mirror the situation in vertebrates is not known.

In contrast to PRC2, vertebrate PRC1 comes in multiple flavours (Gil and O’Loghlen 2014). Each of the four core subunits in canonical PRC1 has between two and five versions (Fig. 1b). Some of these have overlapping functions, for example, RING1A and RING1B, which catalyse ubiquitination of histone H2A, can compensate for each other (de Napoles et al. 2004). However, other subunits may confer unique properties on the complex, for example, CBX7 is the primary ortholog present in PRC1 in ESCs, is required for the maintenance of pluripotency, and is downregulated upon differentiation. CBX2, CBX4 and CBX8 are directly repressed by CBX7 and are upregulated upon lineage commitment (Morey et al. 2012; O’Loghlen et al. 2012). Intriguingly, transient recruitment of PRC1 containing CBX8 is required for the transcriptional activation of several differentiation genes (Creppe et al. 2014). In molecular terms, these differences may in part be conferred by the different affinities of the CBX chromodomains for different modified histones (discussed in detail below (Bernstein et al. 2006b)). However, a recent study suggests that these different functions may not be relevant in some lineages (Pemberton et al. 2014). The authors studied genome-wide distribution of PcG orthologs CBX6, CBX7, CBX8, RING1 and RING2 in human fibroblasts, showing that these proteins colocalise at multiple sites; thus, their functions may be redundant rather than protein specific. In the fly, a single subunit (PC) takes the place of CBX in PRC1 throughout development (Simon and Kingston 2013). Again, it may well be that in the fly, the properties rather than the identities of the PC subunit are developmentally regulated, for example, by posttranslational modifications (Niessen et al. 2009). The fly SU(Z)2 protein shares homology with PSC, fulfils similar functions in in vitro assays (Lo et al. 2009) and coimmunoprecipitates with PC when PC is overexpressed (Poux et al. 2001); thus, it may also participate in PRC1 in vivo and modulate its function; however, this has not been addressed in detail.

Finally, in both flies and vertebrates, a class of non-canonical PRC1s has been identified, which lack CBX or PC proteins and contain the ubiquitin ligase RING (Fig. 1c) (Farcas et al. 2012; Gao et al. 2012; Lagarou et al. 2008; Sanchez et al. 2007). The vertebrate complexes contain additional subunits and are distinguished by different PCGF orthologs (see Fig. 1c). The complex containing PCGF1 (PRC1.1) also contains the histone H3K36 demethylase KDM2B, whereas other PCGF subunits copurify with other accessory proteins (Gao et al. 2012). Interestingly, PRC1s containing each of these different PCGFs have distinct genomic localisations and enzymatic activities compared to canonical complexes, indicating that each PCGF, or the accessory proteins associated with it, may drive targeting by different mechanisms (Gao et al. 2012). *Drosophila* dRAF is the most similar to vertebrate PRC1.1 and consists of dRING, PSC and the histone H3K36 demethylase dKDM2 (Lagarou et al. 2008).

What do these differences in complex diversity tell us about the tasks of PREs in flies and vertebrates? The increased combinatorial potential of mammalian PRC1 compared to its fly counterparts suggests that mammalian PREs would need to be able to contend with a much larger number of unique complexes, with varying subunit compositions and properties during development, potentially requiring different recruitment mechanisms. The fact that vertebrate complexes with different subunit compositions are distributed differently across the genome, and are developmentally regulated, strongly suggests that they may have different preferences for the underlying DNA sequences. Once recruited to a given PRE, the differences in enzymatic properties of various vertebrate complexes may have a profound effect on quantitative properties of the PRE, such as the stability of silencing and the ability to switch between active and silent states.

### Molecular mechanisms of activation and silencing: highly conserved, with a few striking exceptions

The molecular mechanisms of PcG/TrxG-mediated silencing and activation have been covered in detail by several recent reviews (Kingston and Tamkun 2014; Lanzuolo and Orlando 2012; Simon and Kingston 2013). To inform our discussion of how mammalian PREs might compare to those of the fly, we present a parallel analysis of the molecular properties of the vertebrate and fly proteins (Table 1). With few exceptions, most of the molecular properties of PcG and TrxG proteins have indeed been demonstrated for both the fly and the vertebrate counterparts.

**Table 1:** Evidence for common molecular mechanisms mediated by *Drosophila* and vertebrate PcG and TrxG proteins

Molecular mechanism	Vertebrate	Fly
(A) PcG: self-reinforcing mechanisms		
PRC2 trimethylates H3K27 through SET domain of EZH2 /E(Z)	(Cao et al. 2002) (Kuzmichev et al. 2002)	(Czermin et al. 2002) (Muller et al. 2002)
PRC2 binds H3K27me3 via EED/ESC	• (Hansen et al. 2008) (Margueron et al. 2009) • Binding to H3K27me3 stimulates PRC2 HMTase activity (Margueron et al. 2009)	Indirect: mutations in conserved residues of ESC cause developmental defects in *Drosophila* (Margueron et al. 2009)
PRC1 binds H3K27me3 via CBX/PC chromodomain	Different CBX chromodomains have different preferences (Bernstein et al. 2006b) (see also Fig. 2b)	in vitro: (Fischle et al. 2003; Min et al. 2003) in vivo*:* (Wang et al. 2004b)
PRC1 can bind chromatin independently of H3K27me3 in vivo	• PRC1 proteins are recruited to the inactive X chromosome in the absence of EED (Schoeftner et al. 2006). • PRC1 recruited by REST/RUNX1 independent of PRC2 (Dietrich et al. 2012; Yu et al. 2012).	In cultured cells, PC binds the Ubx promoter independently of PHO and E(Z) (Wang et al. 2004b).
PRC1 catalyses monoubiquitylation of H2A on K119 (K118 in fly) through RING1A/1B/dRING	(Cao et al. 2005; de Napoles et al. 2004; Wang et al. 2004a)	(Scheuermann et al. 2012; Wang et al. 2004a)
H2A Ub colocalises with poised polymerase (ser5P)	In mouse ESCs (Brookes et al. 2012) (Stock et al. 2007)	Polycomb colocalises with stalled promoters in *Drosophila* embryos (Enderle et al. 2011) .
PRC2 binds H2A Ub via Aebp2 and Jarid2 in vitro	• Extracts from mouse ESCs (Kalb et al. 2014) • H3K27 methylation activity of human PRC2 containing Jarid2 and Aebp2 is higher on chromatin substrates containing H2Aub than on unmodified substrates (Kalb et al. 2014).	Extracts from *Drosophila* embryos (Kalb et al. 2014)
PRC1 blocks the assembly of transcriptional components.	Reconstituted PRC1 prevents assembly of preinitiation complex in vitro (Lehmann et al. 2012)	Indirect: PcG reduces accessibility to PolII in *Drosophila* embryos (Fitzgerald and Bender 2001; McCall and Bender 1996)
PRC1 compacts chromatin	• In vitro: Different proteins in mouse and fly PRC1 mediate compaction (Grau et al. 2011) (see also Fig. 2a) • In vivo: (Endoh et al. 2012; Eskeland et al. 2010)	• In vitro (Francis et al. 2004) • In vivo: Indirect: PcG reduces chromatin accessibility (Fitzgerald and Bender 2001; McCall and Bender 1996)
Compacted chromatin stimulates PRC2 activity in vitro	• (Yuan et al. 2012) • Correlation between high nucleosome density in H3K27me3 levels in vivo (Yuan et al. 2012)	(Yuan et al. 2012)
PH and PSC SAM domains form homopolymers and heteropolymers	• (Kyba and Brock 1998). • hPH self aggregation regulated by O-GlcNAcylation (Gambetta and Muller 2014)	• (Kim et al. 2002, 2005; Kyba and Brock 1998; Robinson et al. 2012). • PH self -aggregation regulated by O-GlcNAcylation (Gambetta and Muller 2014)
(B) TrxG: self-reinforcing mechanisms		
ASH1L/ASH1 SET domain methylates H3K36 (mono and dimethylation).	• (Tanaka et al. 2007) (Yuan et al. 2011). • Structure of human ASH1L (An et al. 2011).	(Tanaka et al. 2007)
ASH1 required for TRX recruitment	Indirect: Vertebrate ASH1L shows similar distribution to MLL (Gregory et al. 2007)	*Drosophila* ASH1 interacts with TRX, required for TRX recruitment. (Rozovskaia et al. 1999).
MLL/TRX monomethylates H3K4	(Tie et al. 2014) MLL1 and 2 monomethylate H3K4	(Tie et al. 2014) TRX and TRR monomethylate H3K4
Trx interacts with CBP		(Tie et al. 2009, 2014)
CPB acetylates H3K27	p300 and CBP (Pasini et al. 2010b)	(Tie et al. 2009) (Tie et al. 2014)
BRD4/FSH(1) binds acetylated lysine	(Devaiah et al. 2012)	(Kellner et al. 2013). Indirect: colocalisation of fsh(1) S isoform with acetylated histones.
BRD4 phosphorylates Pol II Cter (ser2P)- may promote elongation.	(Devaiah et al. 2012)	No data found. FSH shares homology with BRD4 and may also be a PolII kinase.
(C) PcG and TrxG: antagonistic mechanisms		
KDM2B/dKDM2 demethylates Ash1 mediated H3K36 and promotes H2A ubiquitination via RING1/dRING	• KDM2B is a H3K36 demethylase (He et al. 2008) • Recruits RING1B and promotes H2A Ub (Farcas et al. 2012; Sanchez et al. 2007) (Kyba and Brock 1998; Wu et al. 2013)	(Lagarou et al. 2008)
H3K4 and H3K36 methylation inhibit PRC2	(Yuan et al. 2011) (Schmitges et al. 2011)	(Yuan et al. 2011) (Schmitges et al. 2011)
H3K27Ac antagonises PRC2	(Jung et al. 2010) (Pasini et al. 2010a, b)	(Tie et al. 2012, 2014)

Column 1: molecular mechanism. Complex names (PRC1 or 2: Polycomb repressive complex 1 or 2). Individual protein names (vertebrate/ fly) are given. Columns 2 and 3: vertebrate, fly. If evidence for the mechanism exists, the reference is given, with notes where appropriate. A blank indicates that no references were found. Self-reinforcing PcG-mediated silencing mechanisms (A), self-reinforcing TrxG-mediated mechanisms (B), and those that are antagonistic to PcG-mediated silencing (C)

Interestingly, the molecular activities of the PcG and TrxG proteins fall into three main groups, listed separately in Table 1. The first group (Table 1(A)) contains activities of the PcG proteins that tend to reinforce each other and contribute to transcriptional silencing. For example, PRC1 catalyses the monoubiquitination of histone H2A, which can then be bound by PRC2, stimulating its activity towards methylating H3K27, which can in turn recruit both PRC2 and PRC1 (Table 1(A), rows 1–5). The second group of molecular activities are those of the activating TrxG proteins (Table 1(B)), and again, it is becoming clear that many of these activities cooperate and reinforce each other. Thus, for example, the TRX protein recruits the histone acetyltransferase CBP, which acetylates lysine 27 on histone H3 (among other residues), creating a binding platform for the TrxG protein BRD4, which in turn can phosphorylate RNA Polymerase II, converting it to the elongating form (Table 1(B), rows 4–7). Finally, the third group of activities are those in which a PcG-mediated activity directly antagonises that of a TrxG protein, and vice versa (Table 1(C)). Thus, for example, a lysine demethylase associated with a PcG complex removes the methylation at H3K36, which is catalysed by a TrxG protein, and at the same time promotes histone H2A ubiquitination by an associated PcG protein (Fig. 1c, Table 1(C), row 1).

What does this tell us about the properties of the system and how it may work at PREs? Together, these three groups of activities paint a picture of a bistable system, in which intermediate states are unstable, but once an impulse towards activation or silencing has begun, the system has an inherent molecular momentum that will tend to push it stably towards one or other state. Although several of these mechanisms have been elucidated in vitro and their in vivo relevance remains to be determined, the important feature for our discussion of fly versus mammalian PREs is that these properties appear remarkably conserved. Indeed, the similarity in function is highlighted by several studies showing that mouse PcG proteins can substitute for those of the fly in vivo (Atchison et al. 2003; Laible et al. 1997; Muller et al. 1995).

**Fig. 2:**
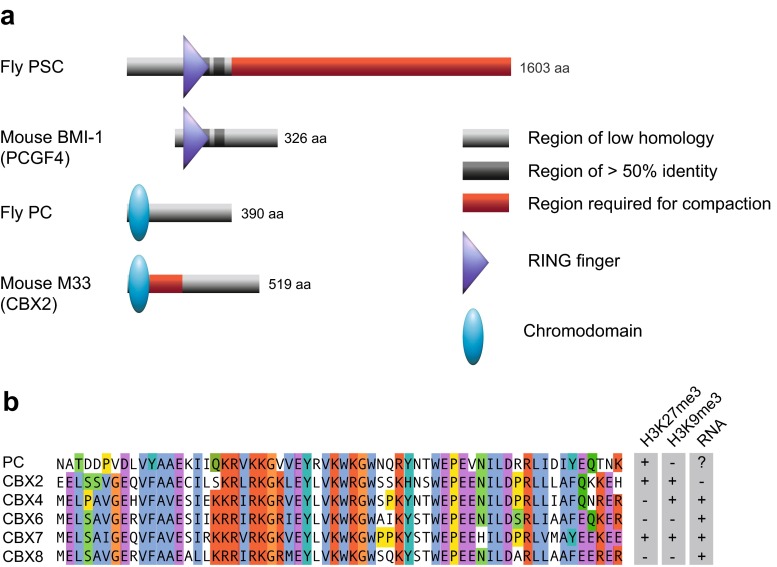
Evidence for different molecular mechanisms mediated by *Drosophila* and vertebrate PcG proteins. **a** Different proteins of the PRC1 complex mediate chromatin compaction in *Drosophila* and mouse (Grau et al. 2011). Purified PRC1 (see Fig. 1) from both fly and mouse can compact nucleosomal arrays in vitro; however, a different protein mediates this activity in the two species. Fly and mouse homologs of the proteins involved are shown. *Red regions* show domains required for compaction in each case, which are overrepresented in basic amino acids. Other domains and degree of conservation between mouse and *Drosophila* are indicated. **b** Alignment of the chromodomains of *Drosophila* Polycomb (PC, amino acids 15–77) and five mouse homologs (CBX, amino acids 1–62) redrawn from Bernstein et al. (2006b) and coloured according to the ClustalX colour scheme http://www.jalview.org/help/html/colourSchemes/clustal.html. On the right of the alignment, in vitro binding preferences of the different chromodomains from Bernstein et al. (2006b) are shown. Histone binding was addressed using modified peptides, Kds ranged between 12 and 49 μM. RNA binding was non-sequence specific. RNA-binding activity of the *Drosophila* PC chromodomain has not been reported to our knowledge

However, a closer look reveals several intriguing differences between flies and vertebrates that may be fundamentally important for determining the quantitative properties of the system. For example, PcG proteins carry multiple posttranslational modifications, many of which are on non-conserved residues (Kaneko et al. 2010; Niessen et al. 2009). These in turn offer opportunities for regulation, suggesting that quantitative properties of specific proteins (for example, enzymatic activities or binding affinities) may be differently regulated in flies and vertebrates. Two further examples are illustrated in Fig. 2. The PRC1 complex can compact chromatin, thus limiting access to remodelling factors and preventing transcriptional activation (see Table 1, row 9). A recent study (Grau et al. 2011) reports the intriguing finding that different proteins within the mouse and fly PRC1 complexes are responsible for mediating this compaction (Fig. 2a); thus, the extent of compaction may be quantitatively different in vivo and may again be subject to very different regulation via posttranslational modifications.

Another important example of quantitative differences between mouse and fly proteins is in the affinity of the chromodomain of the Polycomb protein (Pc, CBX in mammals) for different modified histone H3 tails (Fig. 2b). Although the preference of the fly PC chromodomain for H3K27me3 over H3K9me3 (Fischle et al. 2003; Min et al. 2003) is often assumed to be a major driving force for targeting, the vertebrate CBX proteins show no such preference in vitro (Bernstein et al. 2006b). Indeed, several CBX chromodomains bind equally well to both H3K9me3 and H3K27me3, and one (CBX4) shows a preference for H3K9me3 (Bernstein et al. 2006b) (Fig. 2b). Several, but not all, CBX chromodomains also bind to RNA (Bernstein et al. 2006b; Yap et al. 2010). The choice of CBX subunit for inclusion in PRC1 has a profound effect on the properties of the complex in vivo (Bernstein et al. 2006b; Creppe et al. 2014; O’Loghlen et al. 2012), and these differences may in part be mediated by the different properties of CBX chromodomains.

In summary, the qualitative properties of the system in terms of activation, silencing and switching appear to exist in flies and vertebrates. However, the examples discussed above show that quantitative parameters may be very differently regulated, potentially giving a different output of the system in terms of its effect on transcriptional regulation in specific cases. The extent to which these activities and their regulators are recruited to specific sites will depend in turn on the properties of the PRE.

## Fly and vertebrate PcG target genes: *Hox* regulation is fundamentally different

The fly and mammalian genomes share several hundred PcG targets genes in common (reviewed in Ringrose 2007). Does similar gene identity and function imply a similar role for PcG proteins in their regulation, and thus similar tasks for their PREs? The best-characterised targets of PcG regulation in both flies and vertebrates are the *Hox* genes, which specify the identity of segments along the anterior-posterior axis of the developing embryo. In bilateral animals, including flies and vertebrates, the linear arrangement of *Hox* genes in the *Hox* complexes corresponds to the pattern in which they are expressed along the body axis of the animal, a phenomenon known as colinearity (Duboule 2007; Duboule and Morata 1994). Because of these striking similarities, one might expect that the PREs of the *Hox* genes perform analogous functions in flies and vertebrates. However, as pointed out and discussed in detail by Duboule (2007), several common assumptions about the similarity of fly and vertebrate *Hox* complexes are in fact erroneous. Here, we consider the implications of these differences for the *Hox* PREs.

**Fig. 3:**
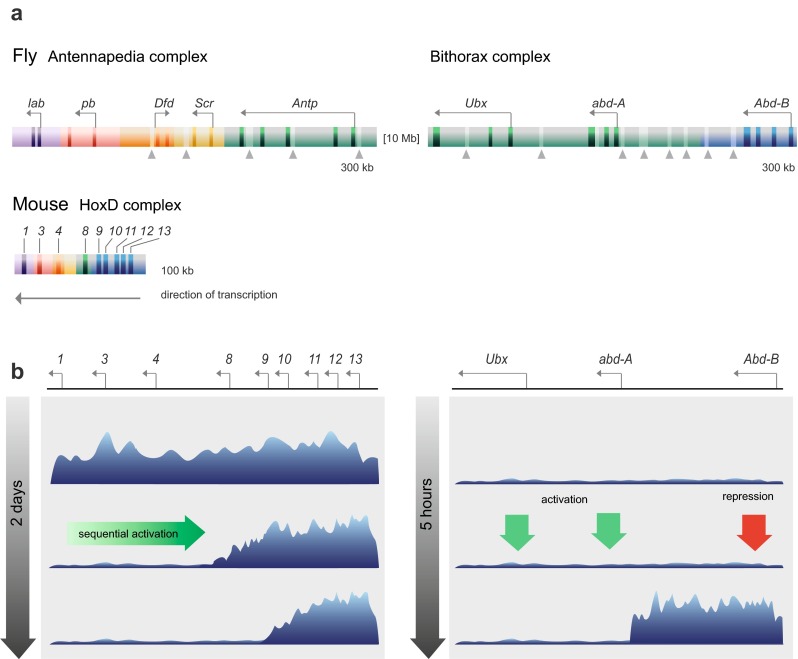
Similarities and differences in *Drosophila* and vertebrate Hox gene regulation. **a** The *Drosophila* Antennapedia (ANT-C) and Bithorax (BX-C) complexes and the mouse HoxD complex are drawn approximately to scale, based on Duboule (2007) and Maeda and Karch (2009). *Dark bars* indicate exons (introns not shown for HoxD due to scaling); *light bars* and *vertical arrowheads* in ANT-C and BX-C indicate experimentally verified PREs (Ringrose and Paro 2004 and references therein). Genes and regulatory regions with a common colour are most closely related in sequence between fly and mouse, and thus belong to the same paralogy group (Duboule 2007). Note that the colour coding is not intended to reflect the different regulatory regions of ANT-C and BX-C as in Maeda and Karch (2009). **b** Pattern of histone H3 lysine 27 methylation at mouse HoxD (*left*) and fly BX-C (*right*) in specific tissues over developmental time. *Left*: summary of data from Soshnikova and Duboule (2009b). In embryonic stem cells, H3K27me3 covers the entire HoxD locus (*top*). In tail buds of E8.5 embryos (*middle*) and E9.5 embryos (*bottom*), Hox genes are sequentially activated leading to clearing of H3K27me3 from the locus. *Right*: summary of data from Bowman et al. (2014) and Maeda and Karch (2009). In early (0–2 h) embryos (*top*), the BX-C very probably lacks H3K27me3 and PcG proteins, based on indirect evidence ((Orlando et al. 1998; Petruk et al. 2012); see main text for details). In parasegment 7 of stage 5 (2–3 h) embryos (*middle*), appropriate Hox genes are activated and repressed by the gap and pair rule gene products (Maeda and Karch 2009). In the same parasegment of later (post 5 h) embryos, repressed domains gain H3K27me3 (Bowman et al. 2014)

The first striking difference is the relative size of the complexes in flies and vertebrates (Duboule 2007). Figure 3a shows the fly *Hox* complexes (ANT-C and BX-C) drawn to scale above the mouse *HoxD* complex, which is far more compact (about 6-fold shorter). It is immediately clear that although the relative order of paralogous genes is conserved, the space occupied both by the transcription units and by intergenic DNA is far greater in the fly than in the mouse. This has implications not only for the number of PREs that can exist within a given regulatory region (see light bars and arrowheads on fly complexes) but also for the distance over which they must communicate with their associated gene promoter.

A further striking difference between mouse and fly development is in the timing of segmentation. In the mouse, segments are sequentially added from anterior to posterior of the developing embryo over a matter of days, accompanied by the sequential activation of *Hox* genes along the complex (Soshnikova and Duboule 2009a). In contrast, in the fly, all segments arise simultaneously within a few hours and the *Hox* genes are simultaneously activated or repressed by the products of the gap and pair rule genes within this short time window (Maeda and Karch 2009) (Fig. 3b).

This difference is accompanied by fundamental differences in the behaviour of the PcG proteins during these early stages of development (Fig. 3b). In early development, the mouse *Hox* genes are entirely covered with H3K27 methylation (Soshnikova and Duboule 2009b). Sequential activation of the *Hox* genes leads to sequential removal of H3K27 methylation, culminating in an appropriate pattern for each segment ((Lan et al. 2007; Mazzoni et al. 2013; Soshnikova and Duboule 2009b). In contrast, in the fly, the *Hox* complexes very likely begin life in a naive state: very little H3K27 methylation is detectable in early embryos before *Hox* expression (Petruk et al. 2012), and PcG and TrxG proteins first become robustly detectable on most *Hox* PREs after the first 2 h of embryogenesis (Orlando et al. 1998). Soon after the gene expression state has been set by activators and repressors, the domain in which *Hox* genes must be repressed in a given segment becomes covered with H3K27 methylation (Bowman et al. 2014). In both mouse and fly, the end result is a sharp boundary between active and silent domains of the complex, but the route by which this is achieved is very different, and may have important implications for the required properties of the PREs in each case.

The *Hox* genes are one example of PcG target genes that are well studied in flies and vertebrates. Whether other common target genes also show differences in their mode of PcG regulation remains to be seen.

## Recruitment of PcG proteins to DNA: the fly and the mammal diverge

The DNA sequences underlying PcG- and TrxG-binding sites appear to show little similarity between flies and mammals. In *Drosophila*, PcG and TrxG proteins require specific DNA-binding proteins to target PREs (reviewed in Kassis and Brown 2013; Ringrose and Paro 2007). Several of their recognition motifs are well characterised and are conserved between *Drosophila* species, although the genomic position of PREs and their exact sequence composition is not, indicating that PREs evolve rapidly (Hauenschild et al. 2008) whilst maintaining similar target domains (Schuettengruber et al. 2014). This rapid evolution of PREs may partially explain why mammalian PREs have been elusive. In addition, the DNA-binding proteins involved in recruiting fly PcG and TrxG are only partially conserved in mammals (summarised in Tables 2 and S1) and their contribution in PcG recruitment is currently not fully understood (reviewed in Do sequence-specific DNA-binding proteins recruit mammalian PcG and TrxG proteins? section below). Recent progress has given rise to various alternative models for mammalian PRE design, invoking CpG islands, binding sites for alternative DNA-binding factors, and non-coding (nc) RNAs as potential components of PREs. We review the evidence for each of these models below.

**Table 2:** Fly and vertebrate DNA binding proteins

Species	Protein	Role in PcG or TrxG function	Binding site
a) Proteins with function first defined in fly: vertebrate homologs			
Drosophila	PHO, PHOL	Yes	• PHO, PHOL bind GCCAT ((Brown et al. 2003; Brown et al 1998). • Consensus found in PREs: CNGCCATNDNND (Mihaly et al. 1998).
Vertebrate	YY1	Disputed	• YY1 binds GCCAT with high nM to low M affinity in vitro (Golebiowski et al. 2012) • Longer site found in vivo, GCCGCCATTTTG YY1 binds with higher affinity than to GCCAT (Kim and Kim 2009)
Drosophila	PSQ	Yes	PSQ binds to GA repeats; same motif as GAF (Hodgson et al. 2001; Huang and Chang 2004; Huang et al. 2002)
Vertebrate	No known homolog		
Drosophila	GAF	Yes	• GAF binds to GA repeats; same motif as PSQ (Pedone et al. 1996). • GAF/PSQ sites required in combination with PHO sites for silencing by the BX-C PRE, "bxd" (Kozma et al. 2008).
Vertebrate	mGAF	Unknown	• c-Krox-Th-POK binds to GA repeats in vitro (Matharu et al. 2010) • Also binds intergenic GA repeats in *Hox* genes in vivo (Srivastava et al. 2013).
Drosophila	ZESTE	Yes	Zeste binds consensus YGAGYG (Biggin et al. 1988)
Vertebrate	No known homolog		
Drosophila	SP1/KLF family	Yes	• Sp1/KLF consensus: RRGGYG. • Family member SPPS binds GGGGCG (Brown et al. 2005)
Vertebrate	SP1/KLF family	Unknown	• SP1 consensus KRGGCGKRRY; binds with high affinity to GGGGCGGGGC (Briggs et al. 1986) • Binds site and activates transcription also if CpG methylated (Holler et al. 1988).
Drosophila	GRH	Yes	• Variable. Consensus site defined as ACYGGTT(T) (Mace et al. 2005) • Binding site in BX-C iab-7 PRE: TGTTTTTT (Blastyak et al. 2006). • Grh binds strongly to CAGGTAG and CAGGCAG; weakly to TAGGTAG (Harrison et al. 2010) • Grh binds AAACCGGTTA from Drosophila Ddc promoter (Uv et al. 1994).
Vertebrate	CP2	Yes	• CP2 consensus GCNCNANCCAG (Kim et al. 1990) • CP2 binds weakly to Drosophila site AAACCGGTTA (Uv et al. 1994).
Drosophila	DSP1	Yes	• Binds GAAAA in Fab-7 PRE. • GAAAA site not enriched at Dsp1 ChIP binding sites (Schuettengruber et al. 2009) • May in fact recognise structural features: HMG domains of Dsp1 bind minor groove of DNA without sequence specificity, instead recognizing DNA structural features. Can also distort or bend DNA (Stros 2010).
Vertebrate	HMGB2	Unknown	• Recognises structural features, see above (Stros 2010).
b) Proteins with function first defined in vertebrates: fly homologs			
Vertebrate	JARID 2	Yes	• Jarid2 binds DNA with no sequence specificity (Patsialou et al. 2005). • GCY and AGS repeats found enriched in Jarid2 bound sites (Peng et al. 2009) • In vitro SELEX suggests Jarid 2 has slight bias towards GC rich sequences but no clear specificity. (Li et al. 2010) • Direct evidence that Jarid2 recruits PRC2 via its DNA binding activity is lacking. Jarid2 may recruit PRC2 via binding to H2AUb (Kalb et al. 2014).
Drosophila	JARID 2	Unknown	• DNA binding activity/ specificity of fly Jarid2 has not been evaluated to our knowledge. • Genetic interaction of fly *Jarid2* with PRE transgenes has not been tested.
Vertebrate	AEBP2	Yes	*Binds to various DNA sequences:* • Gel mobility shift: CTT(N) 15-23cagGCC. (Kim et al. 2009) • Binds CCAAT (Sedaghat et al. 2002) (He et al. 1999) • Motif discovery on AEBP2 bound DNA identified GA rich sites (Kim et al. 2009). • Direct evidence that AEBP2 recruits PRC2 via its DNA binding activity is lacking. AEBP2 may recruit PRC2 by binding to H2AUb (Kalb et al. 2014).
Drosophila	JING/ AEBP2	Unknown	• Direct evidence that Drosophila AEBP2 binds DNA and interacts with PRC2 in vivo is lacking. • AEBP2 and Jarid2 may recruit PRC2 by binding to H2AUb (Kalb et al. 2014).
Vertebrate	REST	Yes	• Binds NRSE/RE1 element TTCAGCACCACGGACAGCGCC (Schoenherr and Anderson 1995) • Consensus binding site derived from REST ChIP-seq data NTCAGCACCNNGGACAGCNCC (Jothi et al. 2008)
Drosophila	Charlatan	Unknown	• N- terminal Zn fingers of Charlatan bind NRSE/RE1 element in vitro TTCAGCACCACGGACAGCGCC (Schoenherr and Anderson 1995; Tsuda et al. 2006) • Consensus derived by gel shift assays on *Drosophila* genomic sites BBHASMVMMVCNGACVKNNCC (Tsuda et al. 2006)
Vertebrate	KDM2B (FBXL10)	Yes	• Binds to non methylated CpG dinucleotides via Zf- CxxC domain (Long et al. 2013a). • ZF- CxxC DNA Recognition requires interaction with both major and minor groove, thus recognition in vivo would require nucleosome free DNA (Long et al. 2013a).
Drosophila	dKDM2	Yes	• dKDM2 has a CxxC domain but DNA binding has not been tested. http://flybase.org/reports/FBgn0037659.html
Vertebrate	RUNX1	Yes	• Runx1 binds TGYGGT (Bowers et al. 2010) and references therein.
Drosophila	Lozenge	Unknown	• Lozenge binds TGYGGT (Wildonger et al. 2005) and references therein.

DNA binding proteins that have been shown to play a role in PcG or TrxG regulation in flies and vertebrates are listed. **a**) Proteins whose function in PcG or TrxG regulation was first defined in flies are listed. Each fly protein is followed by the vertebrate homolog, if known. **b**) Proteins whose function in PcG or TrxG regulation was first defined in vertebrate are listed. Each vertebrate protein is followed by the fly homologue, if known. Column 3: Role in PcG or TrxG function. A comment on whether there is evidence for a role in PcG or TrxG regulation is given. Detailed information on the evidence supporting these statements and references are given in the extended version of this table provided as Table S1. Column 4: Binding site. Consensus binding sites are listed, using the IUPAC code for non-conserved nucleotides http://www.bioinformatics.org/sms/iupac.html. R =A/G; Y=C/T; S=G/C; W=A/T; K= G/T; M= A/C; B = C/G/T; D= A/G/T; H = A/C/T; V= A/C/G; N= A/C/G/T

### Are CpG islands PREs?

The idea that CpG islands may in fact be the long-sought vertebrate PREs has recently gained momentum (Farcas et al. 2012; Klose et al. 2013; Ku et al. 2008; Lynch et al. 2012; Mendenhall et al. 2010; Tanay et al. 2007). CpG islands are 1–2-kb regions of elevated G+C content and high density of CpG dinucleotides compared to the rest of the genome. Over evolutionary time, methylation of CpG dinucleotides elsewhere in the genome leads to their eventual depletion, because methylated cytosine tends to mutate to thymine. However, the vast majority of CpG islands escape this DNA methylation, and thus maintain a high density of CpG dinucleotides compared to their relative depletion in the rest of the genome. (Deaton and Bird 2011). In flies, which have little or no DNA methylation, there is no such depletion of CpG dinucleotides. The mouse and human genomes each contain approximately 24,000 CpG islands, 50 % of which map to annotated promoters, with the remainder likely to coincide with unannotated sites of transcriptional initiation (Illingworth et al. 2010; Deaton and Bird 2011). In mouse ESCs, almost all CpG islands coincide with H3K4me3, regardless of their transcriptional status (Bernstein et al. 2006a; Thomson et al. 2010). H3K4 methylation is recruited to CpG islands through the joint action of the Cpf1 protein (Thomson et al. 2010), and the TrxG proteins MLL1 and MLL2, all of which have a ZF-CxxC domain, which binds specifically to unmethylated CpG dinucleotides (Denissov et al. 2014; Hu et al. 2013; Long et al. 2013a). In addition, numerous studies have observed PRC1 and PRC2 proteins and H3K27me3 at approximately 30 % of these H3K4me3 marked CpG islands in ESCs (Bernstein et al. 2006a; Ku et al. 2008). Thus, CpG islands can recruit both PcG and TrxG proteins. Are these “bivalent” CpG islands PREs?

**Fig. 4:**
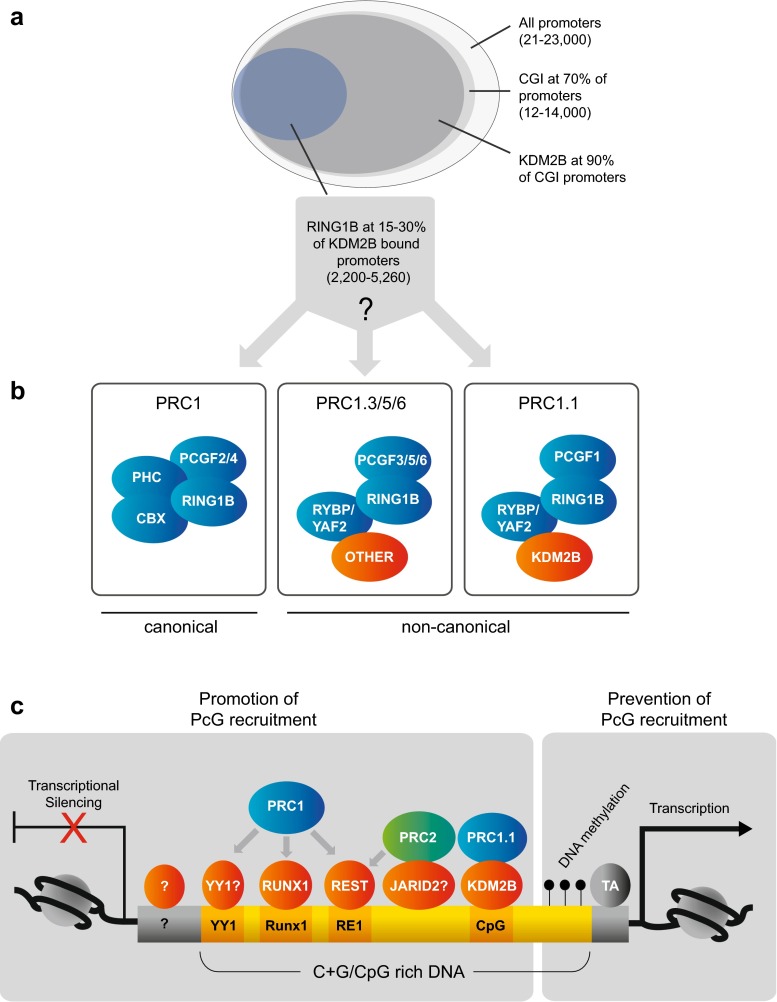
Recruitment of mammalian PcG complexes. **a** Relationship between occurrence of gene promoters, CpG islands, KDM2B and RING1B, according to Deaton and Bird (2011), Farcas et al. (2012), He et al. (2013), and Wu et al. (2013). **b** The RING1B subunit is a component of multiple different complexes, including both canonical and non-canonical PRC1 (Gao et al. 2012) see main text for details. **c** Factors influencing PcG recruitment. A stretch of GC- and CpG-rich DNA is shown (*yellow*). Various motifs for sequence-specific DNA-binding proteins can exist within this DNA (*dark yellow*), and several of these are themselves GC-rich (see Table 2). All of these motifs may also exist in otherwise GC-poor DNA. Proteins that can bind directly to DNA and have been shown or suggested to have role in PcG recruitment are shown in orange. PRC1: indicates all versions of PRC1 except the special case of PRC1.1 which is recruited by KDM2B. *Arrows* indicate that the DNA-binding protein in question does not copurify with the complexes but has been shown to interact by Co-IP. TA: activating transcription factor. See main text for details

Although the correlation of PcG binding with CpG islands is striking, it may to some extent be misleading. Approximately 70 % of annotated gene promoters have a CpG island (Fig. 4a), and many studies have focused on promoter-proximal PcG-binding sites; thus, a correlation of this kind could simply be a consequence of what is found at promoters. Indeed, genome-wide profiling in ESCs has revealed that approximately one quarter to one third of PRC1- and PRC2-binding sites do not map to annotated promoters (Dietrich et al. 2012; Peng et al. 2009) and many of these PcG-bound intergenic sites do not contain CpG islands that conform to computational detection criteria (Dietrich et al. 2012; Hekimoglu-Balkan et al. 2012). On the other hand, computational prediction of GpG islands has been questioned by several recent studies. Illingworth et al. (2010) mapped CpG islands experimentally by CxxC Affinity Purification plus deep sequencing (CAP-seq). The study identified many “orphan” CpG islands that do not map to annotated promoters, are unmethylated in many cell types and are not detected by prediction algorithms. A recent evolutionary study (Long et al. 2013b) compared DNA methylation status across seven vertebrate genomes and found a high conservation of unmethylated DNA at promoters despite varying GC content. Whether these non-methylated islands also correspond to sites of PcG binding was not addressed, but these studies show that the definition of what constitutes a CpG island in vivo is not trivial and raise the issue of whether we should be thinking in terms of “non-methylated islands” rather than “CpG islands”.

Interestingly, in *Xenopus* embryos, sites of H3K27me3 nucleation in early development do not map to promoters, and no correlation between H3K27me3 domains and GC-rich sequences was observed in *Xenopus* or in zebra fish (van Heeringen et al. 2014). Importantly, a strong correlation was found between H3K27me3 and non-methylated DNA, rather than GC richness. Thus, PcG proteins can be recruited in the absence of strong CpG islands, and far from promoters, raising the question of whether the mammalian promoter sites recruit PcG proteins via the DNA sequences that define them as CpG islands, or whether there are other features of these sites such as their non-methylated status, or DNA sequence features other than GC-rich sites, that do the job. The Xenopus study (van Heeringen et al. 2014) strongly suggests that non-methylated status rather than CG richness per se may be essential. In addition, the frequency of CpG dinucleotides in computationally defined CpG islands is typically ten per 100 bases and the total GC content is 65 % (Wachter et al. 2014); thus, there is plenty of room for additional sequence features.

Indeed, several results argue against the necessity and sufficiency of CpG island-like features at the DNA sequence level for PcG recruitment. Several transgenic studies have demonstrated PcG recruitment to ectopic sites in the absence of a CpG island on the transgenic PRE (Schorderet et al. 2013; Sing et al. 2009; Woo et al. 2013). *Xenopus* sequences that recruit H3K27me3 in frog embryos and are GC-poor and unmethylated are able to repress a reporter and recruit H3K27me3 in mouse ESCs (van Heeringen et al. 2014). Furthermore, the deletion of sequences containing CpG islands from the endogenous mouse *HoxD* locus had no effect on the recruitment of H3K27me3 to flanking sites at the locus in vivo (Schorderet et al. 2013).

On the other hand, in support of a role for GC richness and CpG dinucleotides, several studies have shown that insertion of GC-rich sequences at ectopic sites is sufficient to recruit both H3K4me3 and H3K27me3 (Jermann et al. 2014; Lynch et al. 2012; Mendenhall et al. 2010; Wachter et al. 2014). However, below a certain threshold of C+G and CpG dinculeotides, these transgenic sequences become methylated and lose both H3K4me3 and H3K27me3 (Wachter et al. 2014) (Jermann et al. 2014). Further supporting the idea that PcG proteins will bind to GC-rich sites if not blocked by methylation, artificial reduction of endogenous DNA methylation has been shown to lead to a widespread redistribution of both PRC1 and PRC2 to GC-rich sites that were previously methylated (Brinkman et al. 2012; Cooper et al. 2014; Hagarman et al. 2013; Lynch et al. 2012; Reddington et al. 2013). In summary, the available evidence points towards several potential mechanisms of PcG recruitment, which may be complementary and are not mutually exclusive.

Recent mechanistic insights into one of these mechanisms have been provided by the identification of a direct link between one version of PRC1 and its localisation at unmethylated CpG islands. The H3K36 histone demethylase KDM2B (or FBXL10), part of a non-canonical PRC1 (Fig. 1c), specifically recognises non-methylated CpG dinucleotides through its ZF-CxxC domain, and this interaction is required to recruit some PRC1 proteins to a subset of CpG islands (Farcas et al. 2012; He et al. 2013; Long et al. 2013a; Wu et al. 2013), and to prevent DNA methylation (Boulard et al. 2015).

A closer look at the data raises important open questions (Fig. 4a) (Farcas et al. 2012; He et al. 2013; Wu et al. 2013). KDM2B was found to bind to all CpG islands genome-wide, whereas PRC1 components were found at only 15 to 30 % of these sites, raising the question of why PRC1 selects some sites and not others. Furthermore, the ChIP overlaps in Farcas et al. (2012), He et al. (2013) and Wu et al. (2013) were evaluated on the basis of the RING1B protein, which participates in a multitude of different canonical and non-canonical PRC1s, only one of which (PRC1.1) contains the KDM2B protein (Gao et al. 2012) (Figs. 1c and 4b). PRC1.1 binds to distinct sites from other PRC1s and is uniquely characterised by the presence of PCGF1 (and not other PCGFs) and the absence of CBX proteins (Gao et al. 2012) (Fig. 4b). An evaluation of the overlap of RING1B with these subunits would give insights into what proportion of the RING1B bound sites are directly recruited by KDM2B; however, these experiments were not performed in any of the above studies (Farcas et al. 2012; He et al. 2013; Wu et al. 2013). Thus, RING1B may be recruited to many of these sites independently of KDM2B, despite their coincident occurrence. The fact that KDM2B knockdown led to significant loss of RING1B at only 17 % of its targets (Wu et al. 2013) and upregulation of a small number of genes (78 genes of approximately 2000 RING1B bound targets (Farcas et al. 2012)) is consistent with this interpretation.

Nevertheless, these studies identify a direct DNA-based mechanism linking CpG dinucloetides to recruitment of PRC1 members. In future, it will be essential to determine the abundance and developmental regulation of various non-canonical complexes, to understand how they contribute to global targeting. The self-reinforcing nature of many PcG-based mechanisms raises the possibility that a transient recruitment by one complex may be sufficient to trigger a cascade of reactions leading to stable silencing (Blackledge et al. 2014) (Table 1), whose dynamic establishment escapes detection by ChIP profiling. Interestingly, the fly KDM2B homolog, dKDM2, also has a ZF-CxxC domain and participates in a non-canonical PRC1 (dRAF, see Fig. 1c, Tables 2 and S1). The fly genome is abundant in unmethylated CpG dinucleotides; however, the DNA-binding properties and genome-wide distribution of dKDM2 have not been evaluated.

Finally, the extremely intriguing question remains of why mammalian PcG proteins do not bind to all CpG islands, or all sites of KDM2B occupancy. The transgenic studies described above (Jermann et al. 2014; Wachter et al. 2014) clearly demonstrate that recruitment of bivalent chromatin is a default property of unmethylated CpG island-like sequences, raising the question of why all CpG islands do not recruit PcG in vivo, since they are unmethylated and share the sequence features that define CpG islands. Several models have been proposed to account for this discrepancy and fall into two broad, non-mutually exclusive classes. A “chromatin sampling” model proposes that PcG proteins weakly interact with all potential sites, but that transcriptional activity prevents PcG from stably binding potential sites (Klose et al. 2013). Thus, in this model, stable binding is only nucleated in response to a silent promoter, in a similar manner to the proposed mechanism in flies (reviewed in Steffen and Ringrose 2014). Indeed, the occurrence of activating transcription factor motifs or the placement of an active promoter at a CpG island have been shown to be sufficient to block the binding of PRC2 (Caputo et al. 2013; Jermann et al. 2014; Mendenhall et al. 2010; Riising et al. 2014), and global inhibition of transcription leads to a substantial invasion of these silenced sites by PRC2 (Riising et al. 2014). The idea that PcG targeting is merely a result of silencing at permissive CpG islands is neat and simple; however, there are also data that are inconsistent with this model: 30 to 40 % of CpG islands do not acquire PRC2 even after transcriptional inhibition (Riising et al. 2014), and conversely, 10–20 % of active genes do in fact show PcG occupancy is ESCs (reviewed in Ringrose 2007). There must be something more, so what is it?

### Do sequence-specific DNA-binding proteins recruit mammalian PcG and TrxG proteins?

An alternative to this “responsive” model is an “instructive model”, which proposes that sequence-specific DNA-binding proteins recruit PcG and/or TrxG proteins to specific sites, as in the fly (reviewed in Klose et al. 2013). Binding sites for these factors may be embedded in CpG islands and may also occur elsewhere in the genome, giving an additional layer of specificity. These two models are not mutually exclusive, and we propose that their relative contributions at specific genomic sites and developmental stages will be different and that quantitative understanding of these contributions may hold the key to the vertebrate “PRE code”.

**Fig. 5:**
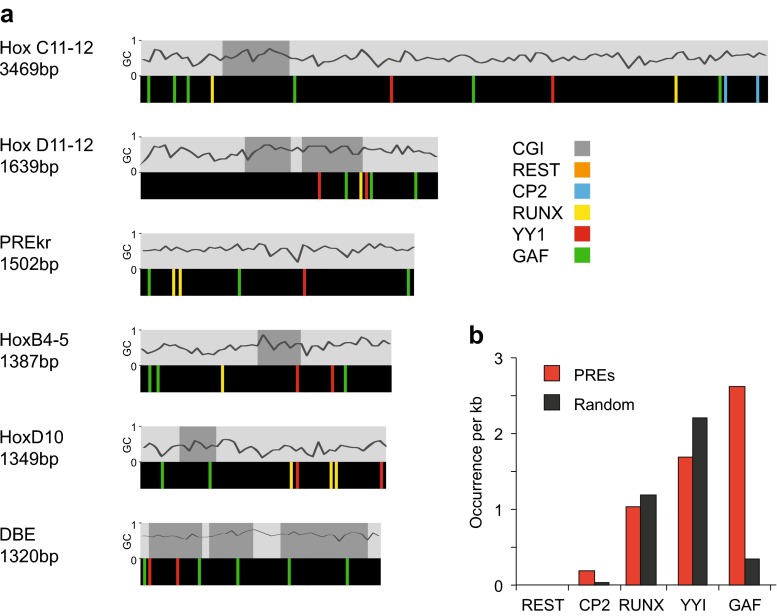
Motif occurrences in mammalian PREs. **a** A selection of mammalian PREs that have been verified to recruit PcG proteins in transgenic assays are shown (see Table 3 for details): HoxC11-12, HoxB4-5 (Woo et al. 2013), HoxD11-12 (Woo et al. 2010), PREkr (Sing et al. 2009), HoxD10 (Schorderet et al. 2013), DBE (Cabianca et al. 2012). Above each element, the % GC is shown, with CpG islands marked in *dark grey*, according to the following criteria: window size 100; minimum length of an island 200; minimum observed/expected CpG 0.6; minimum % GC 50.0. NB with these settings the HoxD10 PRE scores a short 200 bp GpG island; however, this was not detected by the more stringent settings used by Schorderet et al. (2013) and is designated as having no CpG island in Table 3 according to the authors of that study. Motifs for the DNA-binding proteins shown were scored as regular expressions with no mismatch allowance, as follows: REST: NTCAGCACCNNGGACAGCNCC; CP2: GCNCNANCCAG; RUNX:TGYGGT; YY1: GCCAT; GAF: GAGAGA, using the IUPAC code for non-conserved nucleotides as described in the legend to Table 2. **b** Occurrence per kb of motifs in the PREs shown and in random sequence (*black*). To generate random sequence, the total sequence of all elements shown (10.67 kb) was shuffled and searched for motifs. The mean of four iterations is shown

The DNA-binding proteins that have been shown to recruit PcG and TrxG proteins in flies and vertebrates are compared in Tables 2 and S1, and a selection is shown in Fig. 4c. The fly proteins PHO and GAF have mammalian homologs that may play a role in PcG regulation. However, the involvement of YY1 (Pho homolog) with PcG is debated. Whereas the mouse YY1 protein can rescue fly *pho* mutants and repress in flies, no silencing was observed in mammalian cell culture (Srinivasan et al. 2005; Wilkinson et al. 2006). Moreover, genome-wide mapping of PRC1/2 and YY1 showed few overlapping sites in ESCs, rather YY1 sites tend to colocalise with H3K4me3-enriched promoters (Mendenhall et al. 2010; Squazzo et al. 2006). Contrastingly, YY1 sites were required for repression mediated by two transgenic PREs (*HoxD11.12* and *HoxC11.12*) but not at a third (*HoxB4.5*) (Woo et al. 2010, 2013). We note that since YY1 sites contain the motif “GCC”, their mutation may interfere with other proteins that bind GC-rich sequences (Tables 2 and S1). The mammalian homolog of GAGA factor (c-Krox-Th-POK or mGAF) has recently been identified and predicted to bind to the same DNA motif (GAGAG, Table 2) as in flies (Matharu et al. 2010). Profiling of mammalian *Hox* loci revealed intergenic binding sites for mGAF that are enriched for this motif (Srivastava et al. 2013), and the motif is enriched in several mammalian PREs (Fig. 5), but the involvement of mGAF in recruiting PcG proteins has not been investigated.

Several additional proteins have been identified in mammals that have a role in PcG targeting. The transcription factors REST, RUNX1 and E2F6 have been linked to PcG recruitment. E2F6 interacts with and colocalises with non-canonical PRC1s and may recruit these to specific sites (Ogawa et al. 2002; Trimarchi et al. 2001; Trojer et al. 2011). REST and RUNX1 coimmunoprecipitate with PcG proteins, and each occupy a subset of genomic loci bound by PRC1 members on a genome-wide scale. Five percent of REST-binding sites (Dietrich et al. 2012; Ren and Kerppola 2011) and 57 % of RUNX1-binding sites colocalise with PRC1 (Yu et al. 2012). Depletion of REST or RUNX1 leads to decreased PRC1 binding at common binding sites (Dietrich et al. 2012; Yu et al. 2012). Importantly, REST-mediated recruitment of PcG proteins is context dependent: PRC2 depends on REST for its recruitment to GC-rich sequences specifically in neural progenitors and not in ESCs (Arnold et al. 2013), whilst PRC1 can be recruited by REST independently of CpG islands in ESCs (Dietrich et al. 2012). These proteins have fly homologs (Tables 2 and S1), but their connection to PcG function has not been investigated.

In contrast to the above examples, the JARID2 protein is globally required for PRC2 recruitment (Landeira et al. 2010; Li et al. 2010; Pasini et al. 2010a; Peng et al. 2009). JARID2 copurifies and is highly colocalised genome-wide with PRC2 in ESCs (90 % overlap of ChIP-seq peaks (Landeira et al. 2010; Li et al. 2010; Pasini et al. 2010a; Peng et al. 2009). JARID2 can directly bind DNA in vitro without a clear sequence preference; thus, the DNA-binding activity alone is unlikely to add specificity (Tables 2 and S1). Indeed, although JARID2 is required for PRC2 recruitment, direct evidence that this occurs via DNA binding in vivo is lacking (Tables 2 and S1). The recent demonstration that PRC2 containing JARID2 and AEBP2 is able to bind to ubiquitinated histone H2A and that this enhances its HMTase activity, raises the possibility that recruitment involves recognition of existing H2Aub, giving another potential example of a self-reinforcing mechanism (Kalb et al. 2014). This in vitro interaction is conserved for the fly proteins (Kalb et al. 2014); however, fly *Jarid2* mutants do not give *Polycomb* phenotypes (Sasai et al. 2007) and JARID2 does not colocalise highly with PRC2 in flies; thus, whether it has a role in targeting fly PRC2 is unclear (Herz et al. 2012). Consistent with a central role for JARID2 in modulating PRC2 activity, developmentally regulated methylation of mouse JARID2 by EZH2 also increases PRC2 HMTase activity (Sanulli et al. 2015).

Interestingly, mammalian REST, RUNX1, E2F6 and AEBP2 are expressed in specific tissues or developmental stages or at specific times during the cell cycle (Kherrouche et al. 2001) (see Tables 2 and S1). Thus, the recruitment mechanisms that depend on these factors may be required to give a boost of recruitment to relevant targets in specific contexts. This may imply that generally PcG proteins are recruited by other mechanisms. However, it could also imply that we have yet to identify the other DNA-binding factors that specifically target PcG complexes to different classes of targets. This would require either that PcG proteins have acquired specialised functions to recognise a variety of DNA-binding proteins, or that these proteins modulate the accessibility of chromatin to enable access for non-sequence-specific factors such as JARID2.

### Do non-coding RNAs recruit PcG and TrxG proteins? A question of specificity

In *Drosophila* and vertebrates, many PcG target sites are transcribed into ncRNA (Brockdorff 2013; Hekimoglu and Ringrose 2009). This observation, in combination with the fact that several PcG and TrxG proteins can bind to RNA in vitro (Bernstein et al. 2006b; Krajewski et al. 2005), has given rise to the proposal that interactions of PcG and TrxG proteins with specific ncRNAs are responsible for targeting to specific sites in vivo (Cifuentes-Rojas et al. 2014; Kaneko et al. 2014; Zhao et al. 2008). Indeed, several ncRNAs have been shown to be required for PcG and TrxG function in vivo with exquisite specificity (reviewed in Brockdorff 2013; Grossniklaus and Paro 2014; Hekimoglu and Ringrose 2009). However, there are also results that argue against a simple targeting function for ncRNAs. The idea that PcG complexes themselves recognise specific RNA sequence or structural motifs is inconsistent with several observations. First, it is difficult to imagine how a generic protein complex can interact specifically with several hundred different RNA molecules in a pool of several thousand highly abundant other RNA species. Second, several studies have recently shown that PRC2 interacts promiscuously with RNA in vitro (Davidovich et al. 2013) and that this interaction leads to an inhibition of histone-methyltransferase activity of both fly and vertebrate PRC2 (Cifuentes-Rojas et al. 2014; Herzog et al. 2014). It remains to be seen whether other enzymatic activities of the PcG and TrxG complexes are stimulated or inhibited by RNA. A recent study examined Xist RNA using superresolution microscopy and observed that Xist RNA and PcG proteins do not in fact colocalise at high resolution, suggesting that Xist is unlikely to directly recruit PcG proteins to the inactive X-chromosome (Cerase et al. 2014).

A key future challenge will be to reconcile the lack of inherent specificity of PcG and TrxG proteins for specific RNAs in vitro, with the exquisite specificity of some ncRNAs in affecting PcG and TrxG function in vivo. We envisage three non-exclusive models. First, many of the ncRNAs arising from PcG-binding sites are highly developmentally regulated (Dinger et al. 2008; Guttman et al. 2011; Hekimoglu-Balkan et al. 2012; Wang et al. 2011; Herzog et al. 2014). This may offer a means to rapidly modulate PcG and TrxG enzymatic function without affecting recruitment, as observed by Herzog et al. (2014). Second, highly expressed transcripts could represent a decoy for PcG proteins to be displaced from the locus, as proposed by Davidovich et al. (2013) and observed by Herzog et al. (2014). Finally, there may be other specific binding factors that prevent or enable the interaction of certain RNAs with PcG and /or TrxG proteins, as proposed by Herzog et al. (2014). This would enable reversible and regulated switching of the availability of a given RNA to interact with PcG proteins. Although it remains to be seen to what extent these ideas are globally applicable, the important insight from recent work is that ncRNAs are unlikely to be involved in specific targeting, and rather to be involved in modulating the properties of PREs and the proteins bound there.

## Experimental assays for fly and vertebrate PREs

How do we know that a PRE is a PRE? Although genome-wide profiling can give important insights into the localisation and interdependence of chromatin-binding proteins, transgenic reporter assays are essential for defining the DNA sequences that have PRE function.

### Assays in fly

There are several transgenic assays for PRE function that are routinely used in flies. The most commonly used assay is the “miniwhite” reporter, in which transgenic flies are generated, carrying a candidate PRE linked to a minimal promoter driving a reporter gene (miniwhite). The miniwhite reporter is derived from the white gene, which gives a red eye colour in adult flies. Homozygous miniwhite transgenes typically give eye pigment levels of 15 to 50 % of that found in wild-type flies (Okulski et al. 2011). The repressive properties of the PRE, the effects of mutating different motifs and the response of the element to PcG and TrxG mutations are easily scored by quantification of eye pigment levels. The ability of the PRE to recruit PcG and TrxG proteins to the site of the transgene is evaluated by analysis of polytene chromosomes or by ChIP (reviewed in Ringrose and Paro 2004). In addition, fly PREs typically show pairing sensitive silencing (PSS) of miniwhite, whereby the reporter is more strongly silenced in homozygotes (carrying two copies of the transgene) than in heterozygotes (carrying a single copy; Kassis 1994). Recently, it has become possible to perform this assay by integrating different constructs at an identical genomic location, thus enabling a quantitative analysis of the effects of genomic position, to which fly PREs are extremely sensitive (Okulski et al. 2011). Several further assays that also address the ability of the PRE to preserve epigenetic memory of previously established transcriptional states in the absence of the initiating signal have also been devised, showing that several fly PREs can maintain memory of both active and silent states over many cell generations. These assays are more biologically relevant than the miniwhite assay as they address the maintenance of both silencing and activation over developmental time (Simon et al. 1993; Chan et al. 1994; Cavalli and Paro 1998; Perez et al. 2011). The strength of this memory function varies with both the genomic location, the identity of the PRE, and the developmental stage at which the signal to activate or silence is given, indicating that PRE properties are context dependent (reviewed in Steffen and Ringrose 2014).

### Assays in mammalian systems

Several studies that have reported transgenic assays for mammalian PREs or PRE-like sequences are summarised in (Table 3). Comparison of these studies reveals that a wide range of contexts have been used. In contrast to the fly, very few studies have analysed PRE function in living animals (for example, Schorderet et al. 2013; Sing et al. 2009), instead the majority of studies rely on cell culture based assays, allowing rapid evaluation of candidate elements. Given the tractability of these systems, it is surprising that only 9 out of 15 studies assessed the ability of candidate PREs to repress a reporter. Each of these used a different reporter-promoter combination (Table 3). The other studies focused exclusively on the analysis of recruitment of PRC1 and/or PRC2 proteins to putative PREs at ectopic sites (Table 3). A variety of transiently transfected and integrated systems have been used (Table 3). Of the eight studies that used integrated transgenes, only four used targeted integration to compare different elements or variants at the same genomic location, which was a different site in three of the four studies (Arnold et al. 2013; Jermann et al. 2014; Lynch et al. 2012; Riising et al. 2014). Moreover, these studies cover eight different mammalian cell types, and several studies include *Drosophila* reporter assays (Basu et al. 2014; Bengani et al. 2013; Cuddapah et al. 2012; Sing et al. 2009; Vasanthi et al. 2013). Remarkably, studies assessing the binding of TrxG proteins are almost completely lacking. Finally, none of these studies addressed memory properties of mammalian PREs, in terms of testing whether the element can maintain a previously established silent or active state over cell generations in the absence of the initial determining signal. Thus, at present, although there are now a fairly large number of published elements that share some properties of fly PREs, in most cases, it is not possible to draw quantitative comparisons between different elements, and thus, it is very difficult to discern the connection between DNA sequence features and functional properties.

**Table 3:** List of transgenic assays identifying vertebrate PREs

Reference	Element name	Identification of the element	Size	CGI	Transgene type, cell type	Reporter (promoter)	ChIP on transgene (enrichment)	Knockdown
(Sing et al. 2009)	PRE-*kr* (mouse)	Site disrupted by *Kreisler* inversion, which leads to misexpression of MafB gene	3 kb	No	Transient. Mouse F9 embryonic carcinoma cells	LacZ (Hsp68)	BMI1 (+), SUZ12 (+/−)	BMI1 (+), SUZ12 (−)
(Meng et al. 2010)	BREr p16 promoter (human)	BMI1 represses INK4a locus, which encodes p16	210 bp	Yes	Transient. HeLa cells	Luciferase (CMV)	BMI1 (+)	BMI1 (+)
(Woo et al. 2010)	Hox D11.12 PRE (human)	Site in HOXD cluster with low nucleosome occupancy and flanked by PcG binding	1.8 kb	Yes	Transient and random integration. Human mesenchymal stem cells and adipocytes	Luciferase (TK)	BMI1 (+), H3K27me3 (+), SUZ12 (+)	BMI1 (+), EED (+), RYBP (+)
(Mendenhall et al. 2010)	ZFPM2 gene (human)	Locus recruits PRC1/2 and is bivalent.	1.7 kb	Yes	Random integration. Mouse ESCs	No	Ezh2 (+), H3K27me3 (+), H3K4me3 (+), Ring1B (−)	No
(Lynch et al. 2012)	HBA2 gene (human)	α Globin locus recruits PcG proteins and is bivalent in humans but not in mice.	4 kb	Yes	Site-specific integration. Mouse ESCs	No	Cbx7 (+), Ezh2 (+), H3K27me3 (+), H3K4me3 (+)	No
(Cuddapah et al. 2012)	SLC17A7 and Hox A3 PRE (human)	H3K27me3-enriched regions near silenced genes	3.1 kb and 2.8 kb	Yes	Transgenic flies, random integration	*white* (*miniwhite*).	H3K27me3 (+), E(Z) (+), PC (+)	Derepressed in *ph* mutant
(Cabianca et al. 2012)	DBE (human)	Deletion of D4Z4 repeats leads to de-repression of 4q35 genes and loss of PcG	3.3 kb	Yes	Random integration. CHO cells	No	Ring1B (+), Bmi1 (+), H2Aub1 (+), EZH2 (+), SUZ12 (+), H3K27me3 (+)	No
(Vasanthi et al. 2013)	HoxD PRE (mouse)	Known element necessary for setting up the early pattern of Hox gene colinear activation	5 kb	No	Transient. NIH3T3 and HEK293 cells	Luciferase (SV40)	No	No
(Arnold et al. 2013)	Stmn2, Xkr7, Bdnf, Pgbd5 genes (mouse)	REST predicted to recruit PcG proteins. Testing of promoters with REST sites and high CpG content	1.2–2 kb	Yes	Site-specific integration. Mouse ESCs and neural progenitors	No	H3K27me3 (+)	No
(Bengani et al. 2013)	PRE-PIK3C2B (human)	In silico analysis for regions with high density of YY1 consensus motif in the human genome	1 kb	No	Transient. HEK293 cells	GFP (CMV)	No	No
(Woo et al. 2013)	Hox B4.5 and C11.12 PREs (human)	Sites in HOXB/C cluster with low nucleosome occupancy and flanked by PcG protein binding	1.4 kb and 600 bp	No	Transient and random transgenic. Human mesenchymal stem cells	Luciferase (HOXA2)	BMI1 (+), H3K27me3 (+) SUZ12 (+), YY1 (+).	B4.5: BMI1 (+), EED (+), JARID2 (+); C11.12: JARID2 (−)
(Schorderet et al. 2013)	PREd10 (HoxD10) (mouse)	Region necessary for deposition of H3K27me3 at HoxD locus	1.4 kb	No	Random integration. Mouse ESCs	No	H3K27me3 (+), Ring1B (+), Suz12 (+).	No
(Basu et al. 2014)	3L-L and 3L-S DNMT3L gene (human)	CpG island shown to have loss of DNA methylation in cancer samples	486 and 275 bp	Yes	Transient. HEK293 cells	GFP (CMV)	ASHL1 (+), CBX2 (+), EED (+), EZH2 (+), H3K27me3 (+), H3K4me3 (−), MLL (−), PHF1 (+), WDR5 (−)	No
(Riising et al. 2014)	c-Jun promoter (mouse)	Target of PRC2 in ESCs and contains a CpG island.	4 kb and 500 bp	Yes	Site-specific integration. Mouse ESCs and neural progenitors	Luciferase (c-Jun or mPGK)	Suz12 (+)	No
(Jermann et al. 2014)	8 CpG islands (mouse)	Enrichment for H3K27me3 and SUZ12 in genome-wide ChIP datasets from murine ES cells	500 to 900 bp	Yes	Site-specific integration. Mouse ESCs and neural progenitors	Luciferase (Utf1)	H3K27me3 (+), Ring1B (+*), Suz12 (+*)	No
(van Heeringen et al. 2014)	Several sites (Xenopus)	Enrichment for H3K27me3 in Xenopus blastula, and high score in computational prediction	1 kb	No	Transient. Mouse ESCs and *Xenopus* embryos	Luciferase (SV40)	H3K27me3 (+), H3K4me3 (−)	No

Studies identifying vertebrate PREs are listed chronologically. Column 1: authors. Column 2: element name. The name of the identified element and/or genomic location is given. The species in which the element was identified is given in brackets. Column 3: identification of the element. A brief description is given on how the element was identified. Column 4: size of element. Column 5: CpG island. The occurrence of a CpG island in elements is indicated. Column 6: transgene type, cell type. Indicates whether transgenes were evaluated by transient transfection, random or site-specific integration, and in which cell type(s) the transgenes were evaluated. Column 7: reporter. If the effect of the element on reporter gene expression was analysed, the reporter and promoter (in brackets) are given. Column 8: ChIP (enrichment). ChIP enrichment at the transgenic element is given where this was evaluated. (+) indicates strong binding, (+/−) weak binding, (−) no binding (*) differential binding to multiple constructs. Column 9: knockdown. Genes targeted for knockdown are given. (+) indicates de-repression of the reporter upon knockdown, (−) no de-repression

## Computational analysis of fly and vertebrate PREs

**Fig. 6:**
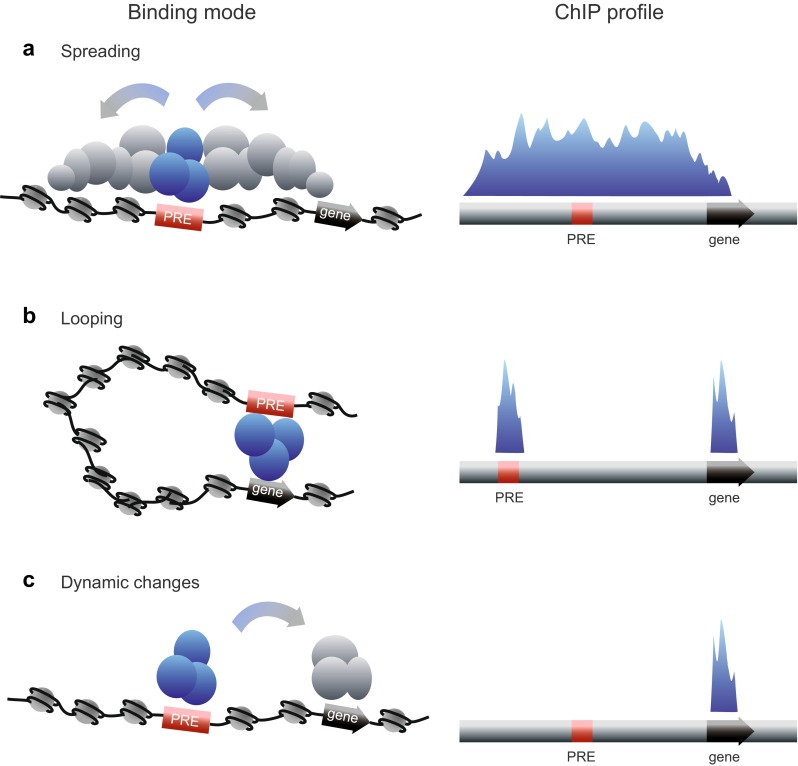
Different modes of PcG binding and their resulting ChIP-binding profiles. On the left are shown different modes of dynamic binding of PcG proteins to PREs. On the right are shown the ChIP profiles that would result from each mode of binding. **a** Spreading. PcG proteins are recruited by a PRE and subsequently spread up and downstream (*left*), resulting in a broad ChIP peak (*right*) from which the PRE is not identifiable. **b** Looping. PcG proteins are recruited by a PRE and subsequently loop to the promoter via higher order interactions (*left*), resulting in two ChIP peaks (*right*) only one of which is a bona fide PRE. **c** Dynamic changes. In the example shown, PcG proteins are recruited by a PRE and are subsequently delivered to a different location (*left*), resulting in a ChIP peak at the site of delivery (in this example, the gene) but not at the site of entry (in this example, the PRE) (*right*). Variations on this theme include different profiles in different cell types, in which only a subset of multiple PREs may be occupied in different tissues or at different times

An alternative for understanding the sequence principles of PREs is computational prediction. Do we still need prediction now that we have genome-wide profiling? Profiling technologies have revolutionised the way we address many questions in gene regulation, allowing the quantification of expression levels of hundreds of genes and the identification of thousands of protein-binding sites in the genome. However, in the search for mammalian PREs, genome-wide profiling has several caveats as shown in Fig. 6, because not every site of protein enrichment or histone modification is likely to be a PRE. Multiple ChIP peaks or large domains may represent both primary recruitment sites, and secondary sites to which the proteins spread or loop after recruitment by PREs. Furthermore, different tissues show different ChIP profiles and no single tissue will give information on all potential sites that can act as PREs.

Computational prediction approaches can be used to complement profiling data, because they generate a model of underlying sequence determinants that can be highly valuable for distinguishing primary from secondary sites, and for identifying sequence characteristics that are not limited by tissue specificity. For example, computational prediction of fly PREs based on known motifs and trained to distinguish verified PREs from non-PREs identified approximately 20 % of ChIP-binding sites that were later observed in *Drosophila* embryos (Ringrose et al. 2003), discussed in (Hauenschild et al. 2008). Besides identifying many new PREs that were not known in 2003, comparison with later profiling datasets enables a classification into three categories: (i) predicted sites that are also ChIP enriched (these sites contain the motifs in question in particular configurations and are enriched in ChIP experiments); (ii) predicted sites that do not contain a ChIP enrichment in the tissue in question (these sites are computationally indistinguishable from class (i)); and (iii) non-predicted sites (those that have a ChIP enrichment but do not contain the motifs used for the prediction). This classification opens the door to formulating relevant questions: for example, why are some predicted sites bound and others not? Why are some bound sites not predicted? Indeed, inclusion of comparative genomic information from different fly species increases the overlap between ChIP and predictions to 34 % (Hauenschild et al. 2008), which is in the range of overlap observed between different ChIP-profiling studies of PcG proteins (28–34 % (Hauenschild et al. 2008) and references therein). Computational prediction of vertebrate PREs is less straightforward since it is currently less clear which DNA-binding proteins or DNA sequence features initiate PcG binding.

Another limitation is imposed by the small number of verified vertebrate PREs and the lack of specific DNA motifs therein. Interestingly, sequence mining of individual vertebrate PREs using fly motifs mainly identified GAF/mGAF and Pho/YY1 motifs (Bengani et al. 2013; Cabianca et al. 2012; Sing et al. 2009; Woo et al. 2010). However, these short motifs occur frequently in any sequence, and their general relevance to vertebrate PREs is not clear. Figure 5 shows an extended analysis of the occurrence of DNA motifs and sequence features discussed above (Do sequence-specific DNA-binding proteins recruit mammalian PcG and TrxG proteins? section) in a selection of verified mammalian PREs. This reveals that although several motifs do recur in vertebrate PREs (Fig. 5a), only the GAF motif occurs more frequently in PREs than in random sequence (Fig. 5b). Furthermore, there are large stretches of sequence in each PRE in which none of the motifs occur, so there may be additional motifs yet to be found. De novo motif discovery of PRC2 genome-wide binding sites further identified overrepresented repeat sequences, for example, GA and GC repeats (Hajjari et al. 2014; Hunkapiller et al. 2012; Kim et al. 2009; Peng et al. 2009). Cross-species analysis of intergenic and intronic *Hox* sequences revealed enrichments of GA repeats and poly-T stretches in comparison to housekeeping gene sequences (Bengani et al. 2007). Analysis of repetitive sequences in H3K27me3 domains in *Xenopus* revealed high enrichments (over 50-fold) of TAGA and TG repeats (van Heeringen et al. 2014). Distinct motifs have been identified in BMI1 (Meng et al. 2010) and AEPB2 peaks (Kim et al. 2009) (see Tables 2 and S1).

Going beyond motif discovery, several computational approaches have identified sequence features that predictively distinguish PcG-binding sites from non-target sites. As discussed above, several studies have shown a strong correlation between CpG islands and PcG binding (Hunkapiller et al. 2012; Ku et al. 2008; Mendenhall et al. 2010; Tanay et al. 2007) and further analysis identified binding sites for known transcription factors able to discern 2/3 of PcG-bound CpG islands from non-bound CpG islands in ESCs (Ku et al. 2008). Binding motifs for REST and SNAIL have been shown to be predictive for dynamic changes in H3K27me3 during neural differentiation (Arnold et al. 2013). The most thorough prediction to date trained a predictive algorithm to distinguish H3K27me3 enriched from non-enriched domains, on the basis of enrichment and depletion of 8-mer motifs (van Heeringen et al. 2014). The algorithm was initially trained on *Xenopus* H3K27 domains, which are relatively GC-poor. Interestingly, the frog-trained algorithm was also able to distinguish human and zebrafish H3K27me3 domains reasonably well. Similar training on human and zebrafish data revealed a large number of 8-mer motifs that are enriched or depleted in all three species, suggesting that despite the difference in GC content, vertebrate PREs may share common sequence principles.

Each of these predictive studies identified sequence features that are able to distinguish PcG targets from non-targets, and evaluated performance by comparing to ChIP enrichments (Arnold et al. 2013; Ku et al. 2008; van Heeringen et al. 2014). It would be interesting in future to evaluate sites predicted using these features that are not already covered by known ChIP enrichments, as these may lead to novel PREs that are not detected in the available ChIP datasets. In future, computational approaches such as these will be of high importance to identify the specific nucleation sites as opposed to those that are created by spreading or looping, to understand the minimal sequence requirements to establish a PcG domain, and to distinguish primary from secondary peaks (Fig. 6).

## Conclusion and perspectives

**Fig. 7:**
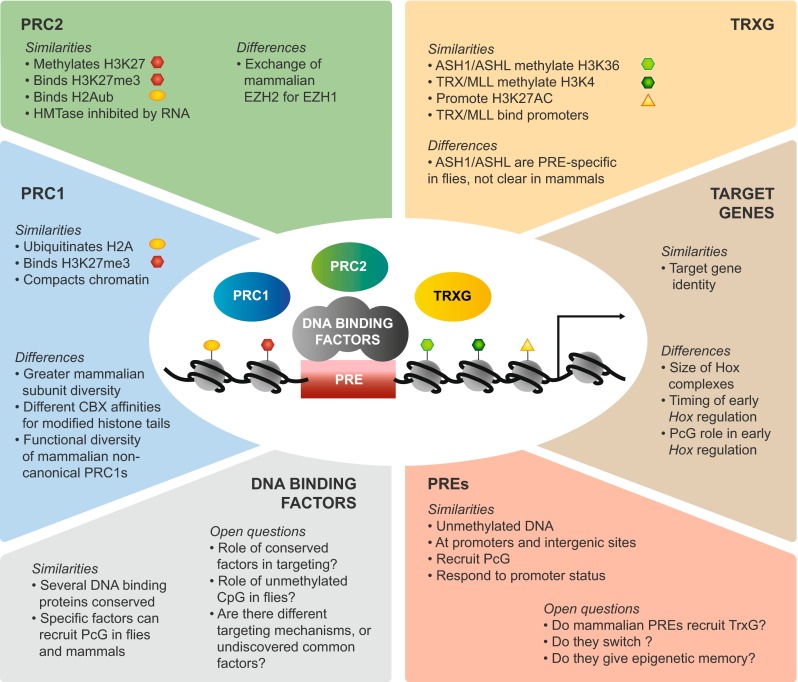
Summary of similarities and differences relevant for fly and vertebrate PREs. The figure summarises the main points of this review. For PRC1, PRC2, TRXG, and target genes, key similarities and differences are listed, discussed in detail in the main text. For DNA-binding factors and PREs, open questions are identified, discussed in the conclusion section of the review

In this review, we initially set out to address three questions, by comparing the protein and nucleic acid components of the PcG and TrxG regulatory system in flies and vertebrates: why are PREs not conserved? Do mammalian PREs use different sequences but perform essentially the same function as fly PREs? Or does mammalian PcG/TrxG regulation play by fundamentally different rules to those in the fly? In conclusion, we find that some of these questions can be answered better than others, and some in fact need to be reformulated. Figure 7 summarises the main findings of this analysis. In considering the properties of the PcG and TrxG protein complexes, remarkable similarities emerge. The most important conserved property is the potential for bistability: multiple self-reinforcing mechanisms for both the PcG and TrxG proteins exist, and opposite mechanisms antagonise each other, giving a potential molecular momentum to each extreme state, whilst destabilising intermediate states (Table 1). The conservation of these properties strongly suggests that the system can function similarly in flies and vertebrates. Whether it does so in specific contexts will depend on the quantitative contribution of forces that push the system towards one or other state.

In answer to the question, “why are PREs not conserved?” we propose that this question needs to be reformulated to ask “do we know whether PREs are conserved?” Due to the large number of open questions regarding PRE sequence requirements in both flies and vertebrates, the answer to this second question is currently “no”. The major difference between fly and vertebrate genomes is the lack of DNA methylation in the former. In vertebrate genomes, CpG islands are oases of unmethylated DNA, allowing PcG and TrxG binding, but do these sites simply present a similar binding platform to vertebrate PcG and TrxG proteins as fly PREs do to the fly proteins? To answer this question, it would be extremely interesting to address the role of conserved ZF-CxxC proteins and of CpG dinucleotides at PREs in the fly. Clarification of the roles of conserved factors such as YY1/PHO, mGAF/GAF, REST/Charlatan, AEBP2 and RUNX1/Lozenge (Tables 2 and S1) will add illumination. Finally, there may be undiscovered targeting factors that bind sequences found in both fly and vertebrate PREs. For example, GT repeat stretches are highly enriched at both vertebrate and fly PcG-binding sites (Ringrose et al. 2003; Schuettengruber et al. 2009; van Heeringen et al. 2014) and these motifs have been shown to be essential for PRE-mediated silencing in the fly (Okulski et al. 2011). We ignore “uninteresting repeats” at our peril.

Finally the third question “do mammalian PREs use different sequences but perform essentially the same function as fly PREs?” cannot be answered on the basis of current evidence. Not only do we not understand the extent of similarity or difference in PRE sequence between flies and vertebrates, but the currently available assays for PRE function in vertebrates fall short of addressing fundamental PRE properties beyond PcG recruitment. In the future, it will be essential to devise assays to address whether and to what extent mammalian PREs are similar to fly PREs, namely, whether they are also TREs, whether they can indeed switch between states, and whether they mediate epigenetic memory. An understanding of the quantitative contribution of DNA sequence, genomic context and developmental signalling to these properties will require quantitative assays in which both recruitment and gene expression are monitored, using constructs integrated at identical genomic locations and which are evaluated at different stages of differentiation. Both the vertebrate and the fly fields may benefit from the use of emerging high-throughput assays (Akhtar et al. 2014; Krebs et al. 2014) to systematically dissect the relationship between PRE sequence, genomic context and function. We suspect that the “PRE code” may not be so different after all.

## Electronic supplementary material

Below is the link to the electronic supplementary material.

Supplementary Table 1 Fly and vertebrate DNA binding proteins: expanded version. This table is identical to Table 2 in the main review but contains additional detail on molecular function in column 3. DNA binding proteins that have been shown to play a role in PcG or TrxG regulation in flies (light green) and vertebrates (dark green) are listed. **a)** Proteins whose function in PcG or TrxG regulation was first defined in flies are listed. Each fly protein is followed by the vertebrate homolog, if known. **b)** Proteins whose function in PcG or TrxG regulation was first defined in vertebrate are listed. Each vertebrate protein is followed by the fly homolog, if known. Column 3: Molecular function. Evidence for a role in PcG or TrxG regulation is given if available. Column 4: Binding site. Consensus binding sites are listed, using the IUPAC code for non-conserved nucleotides http://www.bioinformatics.org/sms/iupac.html. R=A/G; Y=C/T; S=G/C; W=A/T; K=G/T; M=A/C; B=C/G/T; D=A/G/T; H=A/C/T; V=A/C/G; N=A/C/G/T. (DOCX 369 kb)
