# Supplementary material for: The quest for mammalian Polycomb response elements: are we there yet?
Source: Chromosoma. 2015 Oct 9;125:471–96. doi: 10.1007/s00412-015-0539-4 (PMC4901126; doi:10.1007/s00412-015-0539-4)
Supplement: Supplementary file 1 — Fly and vertebrate DNA binding proteins: expanded version. This table is identical to Table 2 in the main review but contains additional detail on molecular function in column 3. DNA binding proteins that have been shown to play a role in PcG or TrxG regulation in flies (light green) and vertebrates (dark green) are listed. a) Proteins whose function in PcG or TrxG regulation was first defined in flies are listed. Each fly protein is followed by the vertebrate homolog, if known. b) Proteins whose function in PcG or TrxG regulation was first defined in vertebrate are listed. Each vertebrate protein is followed by the fly homolog, if known. Column 3: Molecular function. Evidence for a role in PcG or TrxG regulation is given if available. Column 4: Binding site. Consensus binding sites are listed, using the IUPAC code for non-conserved nucleotides http://www.bioinformatics.org/sms/iupac.html. R=A/G; Y=C/T; S=G/C; W=A/T; K=G/T; M=A/C; B=C/G/T; D=A/G/T; H=A/C/T; V=A/C/G; N=A/C/G/T. (DOCX 369 kb) [file 412_2015_539_MOESM1_ESM.docx]

**Supplementary Table 1**

**a) Proteins with function first defined in fly: vertebrate homologs**

| **Species** | **Protein** | **Molecular function** | **Binding site** |
| --- | --- | --- | --- |
| Drosophila | PHO, PHOL | - PHO Recruits PC (and BRM) ([Mohd-Sarip et al, 2002](#_ENREF_55)) - PHO and PHOL proteins recruit PRC2 and are required in different contexts ([Wang et al, 2004](#_ENREF_78)). - PHORC complex (PHO + dSfmbt) required for PcG mediated repression ([Klymenko et al, 2006](#_ENREF_41)). - Crystal structure of PHO + dSFMBT ([Alfieri et al, 2013](#_ENREF_1)). | - PHO, PHOL bind GCCAT (([Brown et al, 2003](#_ENREF_13); [Brown et al, 1998](#_ENREF_16)). - Consensus found in PREs: CNGCCATNDNND ([Mihaly et al, 1998](#_ENREF_54)). |
| Vertebrate | YY1 | - YY1 sites required for repression of transgenic PRE reporters ([Woo et al, 2010](#_ENREF_80); [Woo et al, 2013](#_ENREF_81)). - Human YY1 binds MBT domains similarly to PHO. Crystal structure of YY1 + MBDT1 ([Alfieri et al, 2013](#_ENREF_1)). - YY1 can substitute for Pho in flies ([Atchison et al, 2003](#_ENREF_5)). - YY1 does not colocalise with PRC1 or PRC2 in ESCs ([Mendenhall et al, 2010](#_ENREF_53)). | - YY1 binds GCCAT with high nM to low μM affinity *in vitro* ([Golebiowski et al, 2012](#_ENREF_23)) - Longer site found in vivo, GCCGCCATTTTG YY1 binds with higher affinity than to GCCAT ([Kim & Kim, 2009](#_ENREF_40)) |
| Drosophila | PSQ | - PSQ mutations enhance PcG phenotypes; PSQ copurifies with PcG and GAF ([Hodgson et al, 2001](#_ENREF_29); [Huang & Chang, 2004](#_ENREF_31); [Huang et al, 2002](#_ENREF_32)) - PSQ and GAF interact and are highly colocalised on polytene and mitotic chromosomes ([Schwendemann & Lehmann, 2002](#_ENREF_69)). | - PSQ binds to GA repeats; same motif as GAF ([Hodgson et al, 2001](#_ENREF_29); [Huang & Chang, 2004](#_ENREF_31); [Huang et al, 2002](#_ENREF_32)) |
| Vertebrate | No known homolog |  |  |
| Drosophila | GAF | - ATP-dependent nucleosome remodeling, has many roles outside PcG/TrxG regulation([Becker, 1995](#_ENREF_6)). - Required in embryonic but not larval stages for activation of homeotic genes ([Bejarano & Busturia, 2004](#_ENREF_7)) - Facilitates PcG binding to chromatin in vitro ([Mahmoudi et al, 2003](#_ENREF_51); [Mulholland et al, 2003](#_ENREF_56)) | - GAF binds to GA repeats; same motif as PSQ ([Pedone et al, 1996](#_ENREF_59)). - GAF/PSQ sites required in combination with PHO sites for silencing by the BX-C PRE,"bxd"([Kozma et al, 2008](#_ENREF_42)). |
| Vertebrate | mGAF | - Closest mammalian GAF homolog: c-Krox-Th-POK, encoded by *zbtb7b* ([Matharu et al, 2010](#_ENREF_52)). - *Zbtb3* product is also predicted homolog([Kumar, 2011](#_ENREF_43)). | - c-Krox-Th-POK binds to GA repeats *in vitro* ([Matharu et al, 2010](#_ENREF_52)) - Also binds intergenic GA repeats in *Hox* genes *in vivo* ([Srivastava et al, 2013](#_ENREF_73)). |
| Drosophila | ZESTE | - Zeste binds directly to Ubx promoter ([Biggin et al, 1988](#_ENREF_8)). - Zeste copurifies with PRC1 ([Saurin, 2001](#_ENREF_66)). - Zeste binds reconstituted PRC1, increases transcription inhibition *in vitro*, preference for templates with Zeste binding sites. ([Mulholland et al, 2003](#_ENREF_56)) - Zeste binding sites and Zeste are required for maintenance of Ubx repression. ([Hur et al, 2002](#_ENREF_33)) | Zeste binds consensus YGAGYG ([Biggin et al, 1988](#_ENREF_8)) |
| Vertebrate | No known  homolog |  |  |
| Drosophila | SP1/KLF family | - SPPS (SP1 factor for PSS) enhances PHO mutant phenotype, colocalises virtually identically with PSC on polytene chromosomes, and is required for pairing sensitive silencing of PRE transgenes - ([Brown & Kassis, 2010](#_ENREF_15)) | - Sp1/KLF consensus: RRGGYG. - SPPS binds GGGGCG ([Brown et al, 2005](#_ENREF_14)) |
| Vertebrate | SP1/KLF family | - Link to PcG not explicitely addressed. - Mammalian YY1 and Sp1 interact biochemically and functionally([Lee et al, 1993](#_ENREF_47); [Seto et al, 1993](#_ENREF_71)). | - SP1 consensus KRGGCGKRRY; binds with high affinity to GGGGCGGGGC ([Briggs et al, 1986](#_ENREF_12)) - Binds site and activates transcription also if CpG methylated ([Holler et al, 1988](#_ENREF_30)). |
| Drosophila | GRH | - Grh binds to the iab-7 PRE and interacts genetically and biochemically - with Pho ([Blastyak et al, 2006](#_ENREF_10)).   GRH binds to dRING ([Tuckfield et al, 2002](#_ENREF_76)). | - Variable. Consensus site defined as ACYGGTT(T) ([Mace et al, 2005](#_ENREF_50)) - Binding site in BX-C iab-7 PRE: TGTTTTTT ([Blastyak et al, 2006](#_ENREF_10)). - Grh binds strongly to CAGGTAG and CAGGCAG; weakly to TAGGTAG ([Harrison et al, 2010](#_ENREF_24)) - Grh binds AAACCGGTTA from Drosophila Ddc promoter ([Uv et al, 1994](#_ENREF_77)). |
| Vertebrate | CP2 | - GRH family member CP2 binds to RING1B (DinG). Interaction required for transcriptional repression ([Tuckfield et al, 2002](#_ENREF_76)). - CP2 and GRH DNA binding and dimerisation domain conserved ([Uv et al, 1994](#_ENREF_77)). | - CP2 consensus GCNCNANCCAG ([Kim et al, 1990](#_ENREF_37)) - CP2 binds weakly to Drosophila site AAACCGGTTA ([Uv et al, 1994](#_ENREF_77)). |
| Drosophila | DSP1 | - Role in PcG regulation disputed: Dsp1 mutants show *trxG* phenotypes([Decoville et al, 2001](#_ENREF_18); [Rappailles et al, 2005](#_ENREF_61)). - Dsp1 required for recruitment of PcG to polytene chromosomes, Dsp1 binding sites (GAAAA) required for silencing at Fab-7 transgenic PRE ([Dejardin et al, 2005](#_ENREF_19)). - GAAAA site not required for endogenous bxd PRE silencing ([Kozma et al, 2008](#_ENREF_42)). | - Binds GAAAA in Fab-7 PRE. - GAAAA site not enriched at Dsp1 ChIP binding sites ([Schuettengruber et al, 2009](#_ENREF_68)) - May in fact recognise structural features: HMG domains of Dsp1 bind minor groove of DNA without sequence specificity, instead recognizing DNA structural features. Can also distort or bend DNA ([Stros, 2010](#_ENREF_74)). |
| Vertebrate | HMGB2 | - Connection with Polycomb not studied. HMGB2 can act as an activator of transcription ([Laurent et al, 2010](#_ENREF_46)). | - Recognises structural features, see above ([Stros, 2010](#_ENREF_74)). |

**b) Proteins with function first defined in vertebrates: fly homologs**

| **Species** | **Protein** | **Molecular function** | **Binding site** |
| --- | --- | --- | --- |
| Vertebrate | JARID 2 | - Vertebrate JARID2 is in complex with and colocalises with PRC2 genome wide. Required for PRC2 binding and ESC differentiation ([Landeira et al, 2010](#_ENREF_45); [Li et al, 2010](#_ENREF_48); [Pasini et al, 2010](#_ENREF_57); [Peng et al, 2009](#_ENREF_60); [Shen et al, 2009](#_ENREF_72)) - Jarid2 is methylated by PRC2; methylated Jarid2 stimulates PRC2 mediated methylation of H3K27 ([Sanulli et al, 2015](#_ENREF_64)) | - Jarid2 binds DNA with no sequence specificity ([Patsialou et al, 2005](#_ENREF_58)). - GCY and AGS repeats found enriched in Jarid2 bound sites ([Peng et al, 2009](#_ENREF_60)) - In vitro SELEX suggests Jarid 2 has slight bias towards GC rich sequences but no clear specificity. ([Li et al, 2010](#_ENREF_48)) - Direct evidence that Jarid2 recruits PRC2 via its DNA binding activity is lacking. Jarid2 may recruit PRC2 via binding to H2AUb ([Kalb et al, 2014](#_ENREF_35)). |
| Drosophila | JARID 2 | *Role in Drosophila PcG regulation unclear:*   - *Drosophila* *Jarid2* mutants do not give *Polycomb* phenotypes ([Sasai et al, 2007](#_ENREF_65)). - Fly JARID2 in complex with PRC2, but not highly colocalised ([Herz et al, 2012](#_ENREF_28)) - Fly PRC2 binds H2A Ub via Aebp2 and Jarid2 *in vitro* ([Kalb et al, 2014](#_ENREF_35)) | - DNA binding activity/ specificity of fly Jarid2 has not been evaluated to our knowledge. - Genetic interaction of fly *Jarid2* with PRE transgenes has not been tested. |
| Vertebrate | AEBP2 | - AEBP2 required for optimal enzymatic activity of PRC2 ([Cao & Zhang, 2004](#_ENREF_17)). - ([He et al, 1999](#_ENREF_25)) AEBP2 RNA expressed ubiquitously in mouse tissues. - ([Kim et al, 2011](#_ENREF_38)) AEBP2 expressed in neural crest in developing mouse embryo. AEBP2 -/- knockout is embryonic lethal. - AEBP2 targets partially overlap with SUZ12 in mouse brain tissue ([Kim et al, 2009](#_ENREF_39)) | *Binds to various DNA sequences:*   - Gel mobility shift: CTT(N) 15-23cagGCC. ([Kim et al, 2009](#_ENREF_39)) - Binds CCAAT ([Sedaghat et al, 2002](#_ENREF_70)) ([He et al, 1999](#_ENREF_25)) - Motif discovery on AEBP2 bound DNA identified GA rich sites ([Kim et al, 2009](#_ENREF_39)). - Direct evidence that AEBP2 recruits PRC2 via its DNA binding activity is lacking. AEBP2 may recruit PRC2 by binding to H2AUb ([Kalb et al, 2014](#_ENREF_35)). |
| Drosophila | JING/ AEBP2 | - Jing/AEBP2 expressed in CNS and trachea, homozygous mutants show defects in CNS differentiation ([Sedaghat et al, 2002](#_ENREF_70)) - Fly PRC2 binds H2A Ub via Aebp2 and Jarid2 *in vitro* ([Kalb et al, 2014](#_ENREF_35)) | - Direct evidence that Drosophila AEBP2 binds DNA and interacts with PRC2 in vivo is lacking. - AEBP2 and Jarid2 may recruit PRC2 by binding to H2AUb ([Kalb et al, 2014](#_ENREF_35)). |
| Vertebrate | REST | - NRSF/REST represses neuronal gene transcription in nonneuronal cells ([Schoenherr & Anderson, 1995](#_ENREF_67)) - TF REST copurifies with Cbx proteins in mouse ESCs ([Ren & Kerppola, 2011](#_ENREF_62)) - TF REST interacts with PRC1 and 2 and is required for PRC1 recruitment to a subset of Polycomb regulated neuronal genes. ([Dietrich et al, 2012](#_ENREF_20)) - Reqirement for REST for PRC1 and PRC2 recruitment is context dependent ([Ren & Kerppola, 2011](#_ENREF_62)) ([Dietrich et al, 2012](#_ENREF_20)) - REST binding sites required for recruitment of H3K27me3 to transgenic reporter. ([Arnold et al, 2013](#_ENREF_4)) | - Binds NRSE/RE1 element TTCAGCACCACGGACAGCGCC ([Schoenherr & Anderson, 1995](#_ENREF_67)) - Consensus binding site derived from REST ChIP-seq data NTCAGCACCNNGGACAGCNCC ([Jothi et al, 2008](#_ENREF_34)) |
| Drosophila | Charlatan | - Required in *Drosophila* for initiation of eye development ([Tsuda et al, 2006](#_ENREF_75)) sensory neurons ([Yamasaki et al, 2011](#_ENREF_83)) and maintenance of intestinal stem cells ([Amcheslavsky et al, 2014](#_ENREF_2)). - Involvement in PcG mediated silencing has not been tested. Indirect evidence: H3K27me3 is reduced in coREST mutant follicle cells ([Domanitskaya & Schupbach, 2012](#_ENREF_21)) (coREST is a co-repressor with REST in mammalian cells([Andres et al, 1999](#_ENREF_3))) | - N- terminal Zn fingers of Charlatan bind NRSE/RE1 element *in vitro* TTCAGCACCACGGACAGCGCC ([Schoenherr & Anderson, 1995](#_ENREF_67); [Tsuda et al, 2006](#_ENREF_75)) - Consensus derived by gel shift assays on *Drosophila* genomic sites BBHASMVMMVCNGACVKNNCC ([Tsuda et al, 2006](#_ENREF_75)) |
| Vertebrate | KDM2B (FBXL10) | - KDM2B is a H3K36 histone demethylase ([He et al, 2008](#_ENREF_26)) - KDM2B co purifies with a non canonical PRC1 complex containing RING1B but lacking Cbx proteins (see also Fig.1) ([Farcas et al, 2012](#_ENREF_22); [Sanchez et al, 2007](#_ENREF_63)) ([Wu et al, 2013](#_ENREF_82)) - KDM2B binds to non methylated CpG islands genome wide via Zf-CxxC domain. - At a subset of these (approximately 15%) PRC1 members also present, and depend on KDM2B for recruitment and H2A Ubiquitylation. ([Farcas et al, 2012](#_ENREF_22)) ([He et al, 2013](#_ENREF_27); [Wu et al, 2013](#_ENREF_82)) - KDM2B mediated targeting of PRC1 is required for PRC2 recruitment at a subset of targets, and for mouse development. ([Blackledge et al, 2014](#_ENREF_9))   *Role in ESC differentiation disputed:*   - Depletion of Kdm2b in mESCs induces early differentiation. ([He et al, 2013](#_ENREF_27)) - ESCs lacking Fbxl10 cannot differentiate properly. ([He et al, 2013](#_ENREF_27); [Wu et al, 2013](#_ENREF_82)) | - Binds to non methylated CpG dinucleotides via Zf- CxxC domain ([Long et al, 2013](#_ENREF_49)). - ZF- CxxC DNA Recognition requires interaction with both major and minor groove, thus recognition *in vivo* would require nucleosome free DNA ([Long et al, 2013](#_ENREF_49)). |
| Drosophila | dKDM2 | - dKDM2 is a H3K36 histone demethylase ([Lagarou et al, 2008](#_ENREF_44)) *Specificity disputed:* ([Kavi & Birchler, 2009](#_ENREF_36)) report H3K4 demethylase activity ) - dKDM2 co purifies with a non canonical PRC1 complex (dRAF) containing dRING and PSC but lacking PC. - dKDM2 is required for efficient H2A ubiquitylation by dRING/PSC in vitro - Substantial overlap in genes affects by siRNA of dKDM2 and PcG in Drosophila S2 cells ([Lagarou et al, 2008](#_ENREF_44)) - dKDM2 not required for viability ([Zheng et al, 2014](#_ENREF_85)) | - dKDM2 has a CxxC domain but DNA binding has not been tested. http://flybase.org/reports/FBgn0037659.html |
| Vertebrate | RUNX1 | - Bmi1 binds directly to Runx1/CBFβ TF complex in mouse megakaryoblastic cells (L8057). - ChIP in L8057 cells: 57% of Ring1b sites bound by Runx1; 57% of Runx1 sites bound by Ring1b. Approx. 3,000 genes. - Ring1B binds via Runx1 independent of PRC2. ([Yu et al, 2012](#_ENREF_84)) | - Runx1 binds TGYGGT ([Bowers et al, 2010](#_ENREF_11)) and references therein. |
| Drosophila | Lozenge | No Polycomb connection found. | - Lozenge binds TGYGGT ([Wildonger et al, 2005](#_ENREF_79)) and references therein. |

References

Alfieri C, Gambetta MC, Matos R, Glatt S, Sehr P, Fraterman S, Wilm M, Muller J, Muller CW (2013) Structural basis for targeting the chromatin repressor Sfmbt to Polycomb response elements. *Genes & development* **27**(21)**:** 2367-2379

Amcheslavsky A, Nie Y, Li Q, He F, Tsuda L, Markstein M, Ip YT (2014) Gene expression profiling identifies the zinc-finger protein Charlatan as a regulator of intestinal stem cells in Drosophila. *Development* **141**(13)**:** 2621-2632

Andres ME, Burger C, Peral-Rubio MJ, Battaglioli E, Anderson ME, Grimes J, Dallman J, Ballas N, Mandel G (1999) CoREST: a functional corepressor required for regulation of neural-specific gene expression. *Proc Natl Acad Sci U S A* **96**(17)**:** 9873-9878

Arnold P, Scholer A, Pachkov M, Balwierz PJ, Jorgensen H, Stadler MB, van Nimwegen E, Schubeler D (2013) Modeling of epigenome dynamics identifies transcription factors that mediate Polycomb targeting. *Genome Research* **23**(1)**:** 60--73

Atchison L, Ghias A, Wilkinson F, Bonini N, Atchison ML (2003) Transcription factor YY1 functions as a PcG protein in vivo. *EMBO J* **22**(6)**:** 1347-1358

Becker PB (1995) Drosophila chromatin and transcription. *Semin Cell Biol* **6**(4)**:** 185-190

Bejarano F, Busturia A (2004) Function of the Trithorax-like gene during Drosophila development. *Dev Biol* **268**(2)**:** 327-341

Biggin MD, Bickel S, Benson M, Pirrotta V, Tjian R (1988) Zeste encodes a sequence-specific transcription factor that activates the Ultrabithorax promoter in vitro. *Cell* **53**(5)**:** 713-722

Blackledge NP, Farcas AM, Kondo T, King HW, McGouran JF, Hanssen LL, Ito S, Cooper S, Kondo K, Koseki Y, Ishikura T, Long HK, Sheahan TW, Brockdorff N, Kessler BM, Koseki H, Klose RJ (2014) Variant PRC1 complex-dependent H2A ubiquitylation drives PRC2 recruitment and polycomb domain formation. *Cell* **157**(6)**:** 1445-1459

Blastyak A, Mishra RK, Karch F, Gyurkovics H (2006) Efficient and specific targeting of Polycomb group proteins requires cooperative interaction between Grainyhead and Pleiohomeotic. *Mol Cell Biol* **26**(4)**:** 1434-1444

Bowers SR, Calero-Nieto FJ, Valeaux S, Fernandez-Fuentes N, Cockerill PN (2010) Runx1 binds as a dimeric complex to overlapping Runx1 sites within a palindromic element in the human GM-CSF enhancer. *Nucleic Acids Res* **38**(18)**:** 6124-6134

Briggs MR, Kadonaga JT, Bell SP, Tjian R (1986) Purification and biochemical characterization of the promoter-specific transcription factor, Sp1. *Science* **234**(4772)**:** 47-52

Brown JL, Fritsch C, Mueller J, Kassis JA (2003) The Drosophila pho-like gene encodes a YY1-related DNA binding protein that is redundant with pleiohomeotic in homeotic gene silencing. *Development* **130**(2)**:** 285-294

Brown JL, Grau DJ, DeVido SK, Kassis JA (2005) An Sp1/KLF binding site is important for the activity of a Polycomb group response element from the Drosophila engrailed gene. *Nucleic Acids Res* **33**(16)**:** 5181-5189

Brown JL, Kassis JA (2010) Spps, a Drosophila Sp1/KLF family member, binds to PREs and is required for PRE activity late in development. *Development* **137**(15)**:** 2597-2602

Brown JL, Mucci D, Whiteley M, Dirksen ML, Kassis JA (1998) The Drosophila Polycomb group gene pleiohomeotic encodes a DNA binding protein with homology to the transcription factor YY1. *Mol Cell* **1**(7)**:** 1057-1064

Cao R, Zhang Y (2004) SUZ12 is required for both the histone methyltransferase activity and the silencing function of the EED-EZH2 complex. *Molecular Cell* **15**(1)**:** 57--67

Decoville M, Giacomello E, Leng M, Locker D (2001) DSP1, an HMG-like protein, is involved in the regulation of homeotic genes. *Genetics* **157**(1)**:** 237-244

Dejardin J, Rappailles A, Cuvier O, Grimaud C, Decoville M, Locker D, Cavalli G (2005) Recruitment of Drosophila Polycomb group proteins to chromatin by DSP1. *Nature* **434**(7032)**:** 533-538

Dietrich N, Lerdrup M, Landt E, Agrawal-Singh S, Bak M, Tommerup N, Rappsilber J, Sodersten E, Hansen K (2012) REST-mediated recruitment of polycomb repressor complexes in mammalian cells. *PLoS Genet* **8**(3)**:** e1002494

Domanitskaya E, Schupbach T (2012) CoREST acts as a positive regulator of Notch signaling in the follicle cells of Drosophila melanogaster. *Journal of cell science* **125**(Pt 2)**:** 399-410

Farcas AM, Blackledge NP, Sudbery I, Long HK, McGouran JF, Rose NR, Lee S, Sims D, Cerase A, Sheahan TW, Koseki H, Brockdorff N, Ponting CP, Kessler BM, Klose RJ (2012) KDM2B links the Polycomb Repressive Complex 1 (PRC1) to recognition of CpG islands. *Elife* **1:** e00205

Golebiowski FM, Gorecki A, Bonarek P, Rapala-Kozik M, Kozik A, Dziedzicka-Wasylewska M (2012) An investigation of the affinities, specificity and kinetics involved in the interaction between the Yin Yang 1 transcription factor and DNA. *The FEBS journal* **279**(17)**:** 3147-3158

Harrison MM, Botchan MR, Cline TW (2010) Grainyhead and Zelda compete for binding to the promoters of the earliest-expressed Drosophila genes. *Dev Biol* **345**(2)**:** 248-255

He GP, Kim S, Ro HS (1999) Cloning and characterization of a novel zinc finger transcriptional repressor. A direct role of the zinc finger motif in repression. *J Biol Chem* **274**(21)**:** 14678-14684

He J, Kallin EM, Tsukada Y, Zhang Y (2008) The H3K36 demethylase Jhdm1b/Kdm2b regulates cell proliferation and senescence through p15(Ink4b). *Nature structural & molecular biology* **15**(11)**:** 1169-1175

He J, Shen L, Wan M, Taranova O, Wu H, Zhang Y (2013) Kdm2b maintains murine embryonic stem cell status by recruiting PRC1 complex to CpG islands of developmental genes. *Nat Cell Biol* **15**(4)**:** 373-384

Herz HM, Mohan M, Garrett AS, Miller C, Casto D, Zhang Y, Seidel C, Haug JS, Florens L, Washburn MP, Yamaguchi M, Shiekhattar R, Shilatifard A (2012) Polycomb repressive complex 2-dependent and -independent functions of Jarid2 in transcriptional regulation in Drosophila. *Mol Cell Biol* **32**(9)**:** 1683-1693

Hodgson JW, Argiropoulos B, Brock HW (2001) Site-specific recognition of a 70-base-pair element containing d(GA)(n) repeats mediates bithoraxoid polycomb group response element-dependent silencing. *Mol Cell Biol* **21**(14)**:** 4528-4543

Holler M, Westin G, Jiricny J, Schaffner W (1988) Sp1 transcription factor binds DNA and activates transcription even when the binding site is CpG methylated. *Genes & development* **2**(9)**:** 1127-1135

Huang DH, Chang YL (2004) Isolation and characterization of CHRASCH, a polycomb-containing silencing complex. *Methods Enzymol* **377:** 267-282

Huang DH, Chang YL, Yang CC, Pan IC, King B (2002) pipsqueak encodes a factor essential for sequence-specific targeting of a polycomb group protein complex. *Mol Cell Biol* **22**(17)**:** 6261-6271

Hur MW, Laney JD, Jeon SH, Ali J, Biggin MD (2002) Zeste maintains repression of Ubx transgenes: support for a new model of Polycomb repression. *Development* **129**(6)**:** 1339-1343

Jothi R, Cuddapah S, Barski A, Cui K, Zhao K (2008) Genome-wide identification of in vivo protein-DNA binding sites from ChIP-Seq data. *Nucleic Acids Res* **36**(16)**:** 5221-5231

Kalb R, Latwiel S, Baymaz HI, Jansen PW, Muller CW, Vermeulen M, Muller J (2014) Histone H2A monoubiquitination promotes histone H3 methylation in Polycomb repression. *Nature structural & molecular biology* **21**(6)**:** 569-571

Kavi HH, Birchler JA (2009) Drosophila KDM2 is a H3K4me3 demethylase regulating nucleolar organization. *BMC research notes* **2:** 217

Kim CG, Swendeman SL, Barnhart KM, Sheffery M (1990) Promoter elements and erythroid cell nuclear factors that regulate alpha-globin gene transcription in vitro. *Mol Cell Biol* **10**(11)**:** 5958-5966

Kim H, Kang K, Ekram MB, Roh TY, Kim J (2011) Aebp2 as an epigenetic regulator for neural crest cells. *PLoS One* **6**(9)**:** e25174

Kim H, Kang K, Kim J (2009) AEBP2 as a potential targeting protein for Polycomb Repression Complex PRC2. *Nucleic Acids Res* **37**(9)**:** 2940-2950

Kim J, Kim J (2009) YY1's longer DNA-binding motifs. *Genomics* **93**(2)**:** 152-158

Klymenko T, Papp B, Fischle W, Kocher T, Schelder M, Fritsch C, Wild B, Wilm M, Muller J (2006) A Polycomb group protein complex with sequence-specific DNA-binding and selective methyl-lysine-binding activities. *Genes & development* **20**(9)**:** 1110-1122

Kozma G, Bender W, Sipos L (2008) Replacement of a Drosophila Polycomb response element core, and in situ analysis of its DNA motifs. *Molecular genetics and genomics : MGG* **279**(6)**:** 595-603

Kumar S (2011) Remote homologue identification of Drosophila GAGA factor in mouse. *Bioinformation* **7**(1)**:** 29-32

Lagarou A, Mohd-Sarip A, Moshkin YM, Chalkley GE, Bezstarosti K, Demmers JA, Verrijzer CP (2008) dKDM2 couples histone H2A ubiquitylation to histone H3 demethylation during Polycomb group silencing. *Genes & development* **22**(20)**:** 2799-2810

Landeira D, Sauer S, Poot R, Dvorkina M, Mazzarella L, Jorgensen HF, Pereira CF, Leleu M, Piccolo FM, Spivakov M, Brookes E, Pombo A, Fisher C, Skarnes WC, Snoek T, Bezstarosti K, Demmers J, Klose RJ, Casanova M, Tavares L, Brockdorff N, Merkenschlager M, Fisher AG (2010) Jarid2 is a PRC2 component in embryonic stem cells required for multi-lineage differentiation and recruitment of PRC1 and RNA Polymerase II to developmental regulators. *Nat Cell Biol* **12**(6)**:** 618-624

Laurent B, Randrianarison-Huetz V, Marechal V, Mayeux P, Dusanter-Fourt I, Dumenil D (2010) High-mobility group protein HMGB2 regulates human erythroid differentiation through trans-activation of GFI1B transcription. *Blood* **115**(3)**:** 687-695

Lee JS, Galvin KM, Shi Y (1993) Evidence for physical interaction between the zinc-finger transcription factors YY1 and Sp1. *Proc Natl Acad Sci U S A* **90**(13)**:** 6145-6149

Li G, Margueron R, Ku M, Chambon P, Bernstein BE, Reinberg D (2010) Jarid2 and PRC2, partners in regulating gene expression. *Genes & development* **24**(4)**:** 368-380

Long HK, Blackledge NP, Klose RJ (2013) ZF-CxxC domain-containing proteins, CpG islands and the chromatin connection. *Biochemical Society transactions* **41**(3)**:** 727-740

Mace KA, Pearson JC, McGinnis W (2005) An epidermal barrier wound repair pathway in Drosophila is mediated by grainy head. *Science* **308**(5720)**:** 381-385

Mahmoudi T, Zuijderduijn LM, Mohd-Sarip A, Verrijzer CP (2003) GAGA facilitates binding of Pleiohomeotic to a chromatinized Polycomb response element. *Nucleic Acids Res* **31**(14)**:** 4147-4156

Matharu NK, Hussain T, Sankaranarayanan R, Mishra RK (2010) Vertebrate homologue of Drosophila GAGA factor. *J Mol Biol* **400**(3)**:** 434-447

Mendenhall EM, Koche RP, Truong T, Zhou VW, Issac B, Chi AS, Ku M, Bernstein BE (2010) GC-rich sequence elements recruit PRC2 in mammalian ES cells. *PLoS Genet* **6**(12)**:** e1001244

Mihaly J, Mishra RK, Karch F (1998) A conserved sequence motif in Polycomb-response elements. *Mol Cell* **1**(7)**:** 1065-1066

Mohd-Sarip A, Venturini F, Chalkley GE, Verrijzer CP (2002) Pleiohomeotic can link polycomb to DNA and mediate transcriptional repression. *Mol Cell Biol* **22**(21)**:** 7473-7483

Mulholland NM, King IF, Kingston RE (2003) Regulation of Polycomb group complexes by the sequence-specific DNA binding proteins Zeste and GAGA. *Genes & development* **17**(22)**:** 2741-2746

Pasini D, Cloos PA, Walfridsson J, Olsson L, Bukowski JP, Johansen JV, Bak M, Tommerup N, Rappsilber J, Helin K (2010) JARID2 regulates binding of the Polycomb repressive complex 2 to target genes in ES cells. *Nature* **464**(7286)**:** 306-310

Patsialou A, Wilsker D, Moran E (2005) DNA-binding properties of ARID family proteins. *Nucleic Acids Res* **33**(1)**:** 66-80

Pedone PV, Ghirlando R, Clore GM, Gronenborn AM, Felsenfeld G, Omichinski JG (1996) The single Cys2-His2 zinc finger domain of the GAGA protein flanked by basic residues is sufficient for high-affinity specific DNA binding. *Proc Natl Acad Sci U S A* **93**(7)**:** 2822-2826

Peng JC, Valouev A, Swigut T, Zhang J, Zhao Y, Sidow A, Wysocka J (2009) Jarid2/Jumonji coordinates control of PRC2 enzymatic activity and target gene occupancy in pluripotent cells. *Cell* **139**(7)**:** 1290-1302

Rappailles A, Decoville M, Locker D (2005) DSP1, a Drosophila HMG protein, is involved in spatiotemporal expression of the homoeotic gene Sex combs reduced. *Biology of the cell / under the auspices of the European Cell Biology Organization* **97**(10)**:** 779-785

Ren X, Kerppola TK (2011) REST interacts with Cbx proteins and regulates polycomb repressive complex 1 occupancy at RE1 elements. *Mol Cell Biol* **31**(10)**:** 2100-2110

Sanchez C, Sanchez I, Demmers JA, Rodriguez P, Strouboulis J, Vidal M (2007) Proteomics analysis of Ring1B/Rnf2 interactors identifies a novel complex with the Fbxl10/Jhdm1B histone demethylase and the Bcl6 interacting corepressor. *Mol Cell Proteomics* **6**(5)**:** 820-834

Sanulli S, Justin N, Teissandier A, Ancelin K, Portoso M, Caron M, Michaud A, Lombard B, da Rocha ST, Offer J, Loew D, Servant N, Wassef M, Burlina F, Gamblin SJ, Heard E, Margueron R (2015) Jarid2 Methylation via the PRC2 Complex Regulates H3K27me3 Deposition during Cell Differentiation. *Mol Cell* **57**(5)**:** 769-783

Sasai N, Kato Y, Kimura G, Takeuchi T, Yamaguchi M (2007) The Drosophila jumonji gene encodes a JmjC-containing nuclear protein that is required for metamorphosis. *The FEBS journal* **274**(23)**:** 6139-6151

Saurin A, Shao, Z., Erdjument-Bromage, H., Tempst, P. and Kingston, R. (2001) A Drosophila Polycomb group complex includes Zeste and dTAFII proteins. *Nature* **412:** 655-660

Schoenherr CJ, Anderson DJ (1995) The neuron-restrictive silencer factor (NRSF): a coordinate repressor of multiple neuron-specific genes. *Science* **267**(5202)**:** 1360-1363

Schuettengruber B, Ganapathi M, Leblanc B, Portoso M, Jaschek R, Tolhuis B, van Lohuizen M, Tanay A, Cavalli G (2009) Functional anatomy of polycomb and trithorax chromatin landscapes in Drosophila embryos. *PLoS Biol* **7**(1)**:** e13

Schwendemann A, Lehmann M (2002) Pipsqueak and GAGA factor act in concert as partners at homeotic and many other loci. *Proc Natl Acad Sci U S A* **99**(20)**:** 12883-12888

Sedaghat Y, Miranda WF, Sonnenfeld MJ (2002) The jing Zn-finger transcription factor is a mediator of cellular differentiation in the Drosophila CNS midline and trachea. *Development* **129**(11)**:** 2591-2606

Seto E, Lewis B, Shenk T (1993) Interaction between transcription factors Sp1 and YY1. *Nature* **365**(6445)**:** 462-464

Shen X, Kim W, Fujiwara Y, Simon MD, Liu Y, Mysliwiec MR, Yuan GC, Lee Y, Orkin SH (2009) Jumonji modulates polycomb activity and self-renewal versus differentiation of stem cells. *Cell* **139**(7)**:** 1303-1314

Srivastava S, Puri D, Garapati HS, Dhawan J, Mishra RK (2013) Vertebrate GAGA factor associated insulator elements demarcate homeotic genes in the HOX clusters. *Epigenetics & chromatin* **6**(1)**:** 8

Stros M (2010) HMGB proteins: interactions with DNA and chromatin. *Biochimica et biophysica acta* **1799**(1-2)**:** 101-113

Tsuda L, Kaido M, Lim YM, Kato K, Aigaki T, Hayashi S (2006) An NRSF/REST-like repressor downstream of Ebi/SMRTER/Su(H) regulates eye development in Drosophila. *EMBO J* **25**(13)**:** 3191-3202

Tuckfield A, Clouston DR, Wilanowski TM, Zhao LL, Cunningham JM, Jane SM (2002) Binding of the RING polycomb proteins to specific target genes in complex with the grainyhead-like family of developmental transcription factors. *Mol Cell Biol* **22**(6)**:** 1936-1946

Uv AE, Thompson CR, Bray SJ (1994) The Drosophila tissue-specific factor Grainyhead contains novel DNA-binding and dimerization domains which are conserved in the human protein CP2. *Mol Cell Biol* **14**(6)**:** 4020-4031

Wang L, Brown JL, Cao R, Zhang Y, Kassis JA, Jones RS (2004) Hierarchical recruitment of polycomb group silencing complexes. *Mol Cell* **14**(5)**:** 637-646

Wildonger J, Sosinsky A, Honig B, Mann RS (2005) Lozenge directly activates argos and klumpfuss to regulate programmed cell death. *Genes & development* **19**(9)**:** 1034-1039

Woo CJ, Kharchenko PV, Daheron L, Park PJ, Kingston RE (2010) A region of the human HOXD cluster that confers polycomb-group responsiveness. *Cell* **140**(1)**:** 99-110

Woo CJ, Kharchenko PV, Daheron L, Park PJ, Kingston RE (2013) Variable requirements for DNA-binding proteins at polycomb-dependent repressive regions in human HOX clusters. *Mol Cell Biol* **33**(16)**:** 3274-3285

Wu X, Johansen JV, Helin K (2013) Fbxl10/Kdm2b recruits polycomb repressive complex 1 to CpG islands and regulates H2A ubiquitylation. *Mol Cell* **49**(6)**:** 1134-1146

Yamasaki Y, Lim YM, Niwa N, Hayashi S, Tsuda L (2011) Robust specification of sensory neurons by dual functions of charlatan, a Drosophila NRSF/REST-like repressor of extramacrochaetae and hairy. *Genes to cells : devoted to molecular & cellular mechanisms* **16**(8)**:** 896-909

Yu M, Mazor T, Huang H, Huang HT, Kathrein KL, Woo AJ, Chouinard CR, Labadorf A, Akie TE, Moran TB, Xie H, Zacharek S, Taniuchi I, Roeder RG, Kim CF, Zon LI, Fraenkel E, Cantor AB (2012) Direct recruitment of polycomb repressive complex 1 to chromatin by core binding transcription factors. *Mol Cell* **45**(3)**:** 330-343

Zheng Y, Hsu FN, Xu W, Xie XJ, Ren X, Gao X, Ni JQ, Ji JY (2014) A developmental genetic analysis of the lysine demethylase KDM2 mutations in Drosophila melanogaster. *Mechanisms of development* **133:** 36-53
